# An Attractor-Based Complexity Measurement for Boolean Recurrent Neural Networks

**DOI:** 10.1371/journal.pone.0094204

**Published:** 2014-04-11

**Authors:** Jérémie Cabessa, Alessandro E. P. Villa

**Affiliations:** 1 Neuroheuristic Research Group, Faculty of Business and Economics, University of Lausanne, Lausanne, Switzerland; 2 Laboratory of Mathematical Economics (LEMMA), University of Paris 2 – Panthéon-Assas, Paris, France; 3 Grenoble Institute of Neuroscience, Faculty of Medicine, University Joseph Fourier, Grenoble, France; Universitat Rovira i Virgili, Spain

## Abstract

We provide a novel refined attractor-based complexity measurement for Boolean recurrent neural networks that represents an assessment of their computational power in terms of the significance of their attractor dynamics. This complexity measurement is achieved by first proving a computational equivalence between Boolean recurrent neural networks and some specific class of 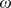-automata, and then translating the most refined classification of 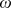-automata to the Boolean neural network context. As a result, a hierarchical classification of Boolean neural networks based on their attractive dynamics is obtained, thus providing a novel refined attractor-based complexity measurement for Boolean recurrent neural networks. These results provide new theoretical insights to the computational and dynamical capabilities of neural networks according to their attractive potentialities. An application of our findings is illustrated by the analysis of the dynamics of a simplified model of the basal ganglia-thalamocortical network simulated by a Boolean recurrent neural network. This example shows the significance of measuring network complexity, and how our results bear new founding elements for the understanding of the complexity of real brain circuits.

## Introduction

In neural computation, understanding the computational and dynamical properties of biological neural networks is an issue of central importance. In this context, much interest has been focused on comparing the computational power of diverse theoretical neural models with those of abstract computing devices. Nowadays, the computational capabilities of neural models is known to be tightly related to the nature of the activation function of the neurons, to the nature of their synaptic connections, to the eventual presence of noise in the model, to the possibility for the networks to evolve over time, and to the computational paradigm performed by the networks.

The first and seminal results in this direction were provided by McCulloch and Pitts, Kleene, and Minsky who proved that first-order Boolean recurrent neural networks were computationally equivalent to classical finite state automata [1]–[3]. Kremer extended these results to the class of Elman-style recurrent neural nets [4], and Sperduti discussed the computational power of different other architecturally constrained classes of networks [5].

Later, Siegelmann and Sontag proved that by considering rational synaptic weights and by extending the activation functions of the cells from Boolean to linear-sigmoid, the corresponding neural networks have their computational power drastically increased from finite state automata up to Turing machines [6]–[8]. Kilian and Siegelmann then generalised the Turing universality of neural networks to a broader class of sigmoidal activation functions [9]. The computational equivalence between so-called “rational recurrent neural networks” and Turing machines has now become standard result in the field.

Following von Neumann considerations [10], Siegelmann and Sontag further assumed that the variables appearing in the underlying chemical and physical phenomena could be modelled by continuous rather than discrete (rational) numbers, and therefore proposed a study of the computational capabilities of recurrent neural networks equipped with real instead of rational synaptic weights [11]. They proved that the so-called “analog recurrent neural networks” are computationally equivalent to Turing machines with advices, hence capable of super-Turing computational power from polynomial time of computation already [11]. In this context, a proper internal hierarchical classification of analog recurrent neural networks according to the Kolmogorov complexity of their underlying real synaptic weights was described [12].

It was also shown that the presence of arbitrarily small amount of analog noise seriously reduces the computational capability of both rational- and real-weighted recurrent neural networks to those of finite automata [13]. In the presence of Gaussian or other common analog noise distribution with sufficiently large support, the computational power of recurrent neural networks is reduced to even less than finite automata, namely to the recognition of definite languages [14].

Besides, the concept of evolvability has also turned out to be essential in the study of the computational power of circuits closer to the biological world. The research in this context has initially been focused almost exclusively on the application of genetic algorithms aimed at allowing networks with fully-connected topology and satisfying selected fitness functions (e.g., performed well on specific tasks) to reproduce and multiply [15]–[18]. This approach aimed to optimise the connection weights that determine the functionality of a network with fixed-topology. However, the topology of neural networks, i.e. their structure and connectivity patterns, greatly affects their functionality. The evolution of both topologies and connection weights following bioinspired rules that may also include features derived from the study of neural development, differentiation, genetically programmed cell-death and synaptic plasticity rules has become increasingly studied in recent years [19]–[26]. Along this line, Cabessa and Siegelmann provided a theoretical study proving that both models of rational-weighted and analog evolving recurrent neural networks are capable of super-Turing computational capabilities, equivalent to those of static analog neural networks [27].

Finally, from a general perspective, the classical computational approach from Turing [28] was argued to “no longer fully corresponds to the current notion of computing in modern systems” [29] – especially when it refers to bio-inspired complex information processing systems. In the brain (or in organic life in general), information is rather processed in an interactive way [30], where previous experience must affect the perception of future inputs, and where older memories may themselves change with response to new inputs. Following this perspective, Cabessa and Villa described the super-Turing computational power of analog recurrent neural networks involved in a reactive computational framework [31]. Cabessa and Siegelmann provided a characterisation of the Turing and super-Turing capabilities of rational and analog recurrent neural networks involved in a basic interactive computational paradigm, respectively [32]. Moreover, Cabessa and Villa proved that neural models combining the two crucial features of evolvability and interactivity were capable of super-Turing computational capabilities [33].

In this paper, we pursue the study of the computational power of neural models and provide two novel refined attractor-based complexity measurement for Boolean recurrent neural networks. More precisely, as a first step we provide a generalisation to the precise infinite input stream context of the classical equivalence result between Boolean neural networks and finite state automata [1]–[3]. Under some natural condition on the type specification of their attractors, we show that Boolean recurrent neural networks disclose the very same expressive power as deterministic Büchi automata [34]. This equivalence allows to establish a hierarchical classification of Boolean neural networks by translating the Wagner classification theory from the Büchi automaton to the neural network context [35]. The obtained classification consists of a pre-well ordering of width 2 and height 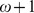 (where 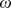 denotes the first infinite ordinal). As a second step, we show that by totally relaxing the restrictions on the type specification of their attractors, the Boolean neural networks significantly increase their expressive power from deterministic Büchi automata up to Muller automata. Hence, another more refined hierarchical classification of Boolean neural networks is obtained by translating the Wagner classification theory from the Muller automaton to the neural network context. This classification consists of a pre-well ordering of width 2 and height 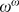. The complexity measurements induced by these two hierarchical classifications refer to the possibility of networks' dynamics to maximally alternate between attractors of different types along their evolutions. They represent an assessment of the computational power of Boolean neural networks in terms of the significance of their attractor dynamics. Finally, an application of this approach to a Boolean model of the basal ganglia-thalamocortical network is provided. This practical example shows that our automata-theoretical approach might bear new founding elements for the understanding of the complexity of real brain circuits.

## Materials and Methods

### Network Model

In this work, we focus on synchronous discrete-time first-order recurrent neural networks made up of classical McCulloch and Pitts cells. Such a neural network is modelled by a general labelled directed graph. The nodes and labelled edges of the graph respectively represent the cells and synaptic connections of the network. At each time step, the status of each activation cell can be of only two kinds: firing or quiet. When firing, a cell instantaneously transmits an action potential throughout all its outgoing connections, the intensity of which being equal to the label of the underlying connection. Then, a given cell is firing at time 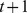 whenever the summed intensity of all the incoming action potentials transmitted at time 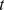 by both its afferent cells and background activity exceeds its threshold (which we suppose without loss of generality to be equal to 1). The definition of such a network can be formalised as follows:

**Definition 1.** *A first-order Boolean recurrent neural network (RNN)* consists of a tuple 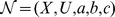, where 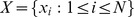 is a finite set of 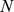 activation cells, 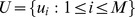 is a finite set of 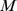 input units, and 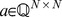, 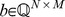, and 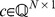 are rational matrices describing the weighted synaptic connections between cells, the weighted connections from the input units to the activation cells, and the background activity, respectively.

The activation value of cells 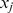 and input units 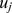 at time 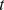, respectively denoted by 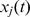 and 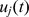, is a Boolean value equal to 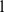 if the corresponding cell is firing at time 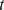 and equal to 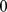 otherwise. Given the activation values 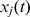 and 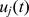, the value 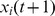 is then updated by the following equation
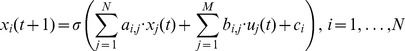
(1)where 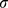 is the classical Heaviside step function, i.e. a hard-threshold activation function defined by 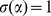 if 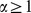 and 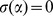 otherwise.

According to Equation (1), the dynamics of the whole network 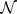 is described by the following governing equation
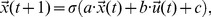
(2)where 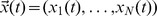 and 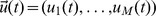 are Boolean vectors describing the spiking configuration of the activation cells and input units, and 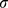 denotes the Heaviside step function applied component by component.

Such Boolean neural networks have already been proven to reveal same computational capabilities as finite state automata [1]–[3]. Furthermore, it can be observed that rational- and real-weighted Boolean neural networks are actually computationally equivalent.

**Figure 1:**
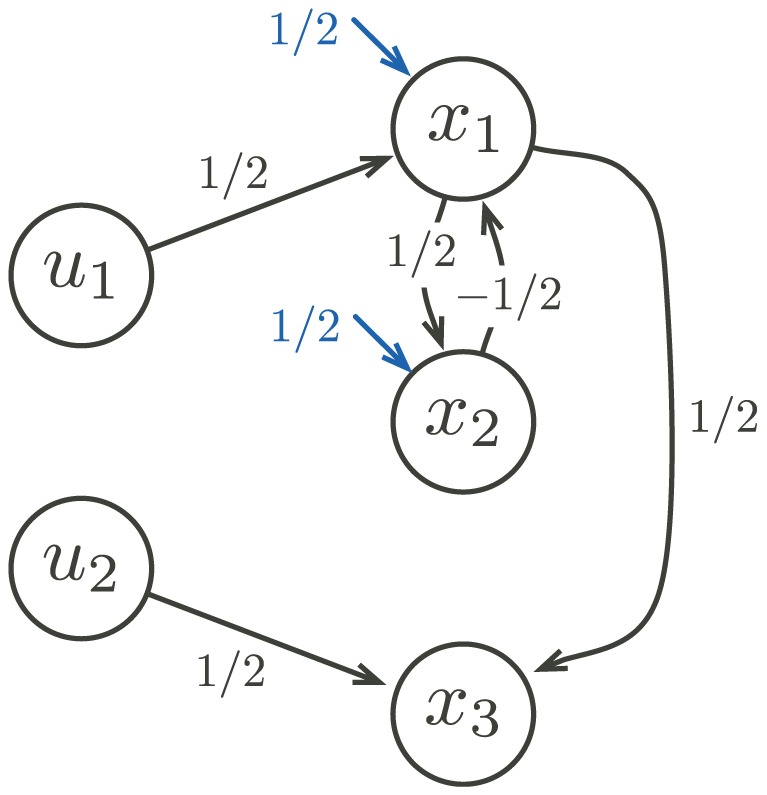
A simple neural network. The network is formed by two input units (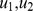) and three activation cells (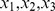). In this example the synaptic weights are all equal to 1/2, with positive sign corresponding to an excitatory input and a negative sign corresponding to a negative input. Notice that both cells 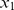 and 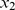 receive an excitatory background activity weighing 1/2.

**Example 1.** Consider the network 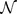 depicted in Figure 1. The dynamics of this network is then governed by the following system of equation:
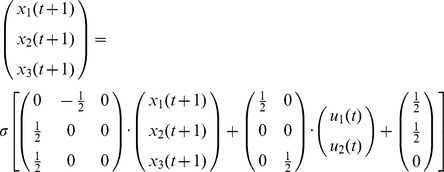



### Attractors

#### Neurophysiological Meaningfulness

In bio-inspired complex systems, the concept of an *attractor* has been shown to carry strong biological and computational implications. According to Kauffman: “Because many complex systems harbour attractors to which the system settle down, the attractors literally are most of what the systems do” [36, p. 191]. The central hypothesis for brain attractors is that, once activated by appropriate activity, network behaviour is maintained by continuous reentry of activity [37], [38]. This involves strong correlations between neuronal activities in the network and a high incidence of repeating firing patterns therein, being generated by the underlying attractors. Alternative attractors are commonly interpreted as alternative memories [39]–[46].

Certain pathways through the network may be favoured by preferred synaptic interactions between the neurons following developmental and learning processes [47]–[49]. The plasticity of these phenomena is likely to play a crucial role to shape the *meaningfulness* of an attractor and attractors must be stable at short time scales. Whenever the same information is presented in a network, the same pattern of activity is evoked in a circuit of functionally interconnected neurons, referred to as “cell assembly”. In cell assemblies interconnected in this way, some ordered and precise neurophysiological activity referred to as preferred firing sequences, or spatio-temporal patterns of discharges, may recur above chance levels whenever the same information is presented [50]–[52]. Recurring firing patterns may be detected without a specific association to a stimulus in large networks of spiking neural networks or during spontaneous activity in electrophysiological recordings [53]–[55]. These patterns may be viewed as *spurious patterns* generated by *spurious attractors* that are associated with the underlying topology of the network rather than with a specific signal [56]. On the other hand, several examples exist of spatiotemporal firing patterns in behaving animals, from rats to primates [57]–[61], where preferred firing sequences can be associated to specific types of stimuli or behaviours. These can be viewed as *meaningful patterns* associated with *meaningful attractors*. However, meaningfulness cannot be reduced to the detection of a behavioural correlate [62]–[64]. The repeating activity in a network may also be considered meaningful if it allows the activation of neural elements that can be associated to other attractors, thus allowing the build-up of higher order dynamics by means of itinerancy between attractor basins and opening the way to chaotic neural dynamics [51], [65]–[70].

The dynamics of rather simple Boolean recurrent neural networks can implement an associative memory with bioinspired features [71], [72]. In the Hopfield framework, stable equilibria of the network that do not represent any valid configuration of the optimisation problem are referred to as *spurious attractors*. Spurious modes can disappear by “unlearning” [71], but rational successive memory recall can actually be implemented by triggering spurious modes and achieving meaningful memory storage [66], [73]–[77]. In this paper, the notions of attractors, meaningful attractors, and spurious attractors are reformulated in our precise Boolean network context. Networks will then be classified according to their ability to alternate between different types of attractive behaviours. For this purpose, the following definitions need to be introduced.

#### Formal Definitions

As preliminary notations, for any 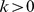, the space of 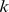-dimensional Boolean vectors is denoted by 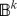. For any vector 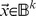 and any 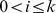, the 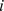-th component of 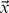 is denoted by 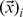. Moreover, the spaces of finite and infinite sequences of 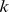-dimensional Boolean vectors are denoted by 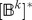 and 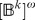, respectively. Any finite sequence of length 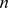 of 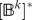 will be denoted by an expression of the form 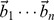, and any infinite sequence of 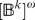 by an expression of the form 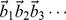, where each 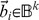. For any finite sequence of Boolean vectors 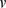, we let the expression 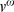 denote the infinite sequence obtained by infinitely many consecutive concatenations of 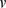, i.e. 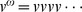.

Now, let 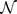 be some network with 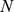 activation cells and 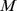 input units. For each time step 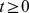, the Boolean vectors 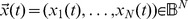 and 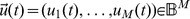 describing the spiking configurations of both the activation cells and input units of 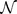 at time 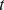 are called the *state* of 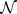 at time 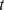 and the *input* submitted to 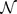 at time 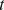, respectively. An infinite *input stream* 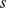 of 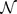 is then defined as an infinite sequence of consecutive inputs, i.e. 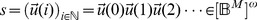. Now, assuming the initial state of the network to be 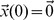, any infinite input stream 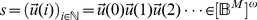 induces via Equation (2) an infinite sequence of consecutive states 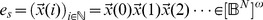 called the *evolution* of 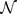 induced by the input stream 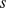.

Note that the set of all possible distinct states of a given Boolean network 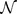 is always finite; indeed, if 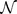 possesses 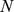 activation cells, then there are at most 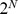 distinct possible states of 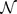. Hence, any infinite evolution 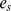 of 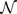 consists of an infinite sequence of only finitely many distinct states. Therefore, in any evolution 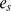 of 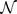, there necessarily exists at least one state that recurs infinitely many times in the infinite sequence 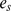, irrespective of the fact that 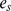 is periodic or not. The non-empty set of all such states that recurs infinitely often in the evolution 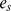 will be denoted by 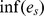.

By definition, every state 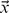 that is visited only finitely often in 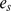 will no longer occur in 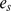 after some time step 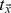. By taking the maximum of these time steps 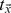, we obtain a global time step 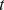 such that all states of 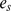 occurring after time 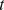 will necessarily repeat infinitely often in 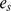. Formally, there necessarily exists an index 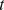 such that, for all 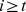, one has 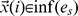. It is important to note that the reoccurrence of the states belonging to 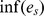 after time step 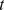 does not necessarily occur in a periodic manner during the evolution 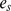. Therefore, any evolution 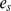 consists of a possibly empty prefix of successive states that repeat only finite many times, followed by an infinite suffix of successive states that repeat infinitely often, yet not necessarily in a periodic way. A set of states of the form 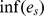 for some evolution 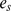 is commonly called an *attractor* of 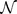 [36]. A precise definition can be given as follows:

**Definition 2.** Let 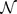 be some Boolean neural network with 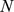 activation cells. A set 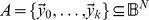 is called an attractor for 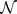 if there exists an input stream 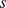 such that the corresponding evolution 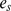 satisfies 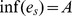.

In other words, an attractor of a Boolean neural network is a set of states such that the behaviour of the network could eventually become forever confined to that set of states. In this sense, the definition of an attractor requires the infinite input stream context to be properly formulated.

In this work, we suppose that attractors can only be of two distinct types, namely either *meaningful* or *spurious*. For instance, the type of each attractor could be determined by its topological features or by its neurophysiological significance with respect to measurable observations associated with certain behaviours or sensory discriminations (see Section “Neurophysiological Meaningfulness” above). From this point onwards, any given network is assumed to be provided with a corresponding classification of all of its attractors into meaningful and spurious types. Further discussions about the attribution of the attractors to either types will be addressed in the forthcoming sections.

An infinite input stream 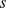 of 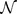 is called *meaningful* if 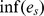 is a meaningful attractor, and it is called *spurious* if 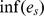 is a spurious attractor. In other words, an input stream is called meaningful (respectively spurious) if the network dynamics induced by this input stream will eventually become confined into some meaningful (respectively spurious) attractor. Then, the set of all meaningful input streams of 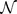 is called the *neural language* of 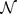 and is denoted by 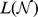. Finally, an arbitrary set of input streams 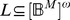 is said to be *recognisable* by some Boolean neural network if there exists a network 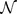 such that 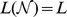.

Besides, if 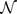 denotes some Boolean neural network provided with an additional specification of the type of each of its attractors, then the *complementary* network 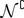 is defined to be the same network as 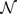 yet with a completely opposite type specification of its attractors. Then, an attractor 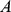 is meaningful for 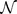 iff 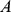 is a spurious attractor for 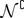 and one has 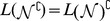. All preceding definitions are illustrated by the next Example 2.

**Example 2.** Let us consider the network 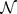 described in Example 1 and illustrated in Figure 1. Let us further assume that the network state where the three cells 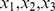 simultaneously fire determines the meaningfulness of the attractors of 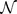. In other words, the meaningful attractors of 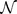 are precisely those containing the state 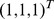; all other attractors are assumed to be spurious.

Let us consider the periodic input stream 
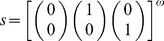
 and its corresponding evolution
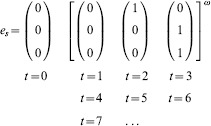
From time step 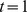, the evolution 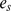 of 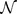 remains confined in a cyclic visit of the states 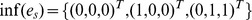. Thence, the set 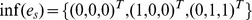 is an attractor of 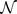. Moreover, since the state 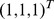 does not belong to 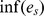, the attractor 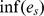 is spurious. Therefore, the input stream 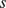 is also spurious, and hence does not belong to the neural language of 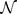, i.e. 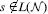.

Let us consider another periodic input stream 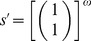 and its corresponding evolution
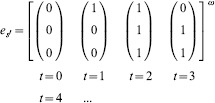
The set of states 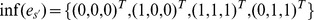 is an attractor, and the evolution 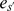 of 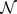 is confined in 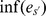 already from the very first time step 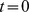. Yet in this case, since the Boolean vector 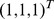 belongs to 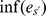, the attractor 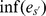 is meaningful. It follows that the input stream 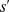 is also meaningful, and thus 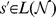.

### 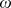-Automata

#### Büchi Automata

A *finite deterministic Büchi automaton* [34] is a 5-tuple 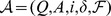, where 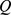 is a finite set called the set of states, 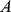 is a finite alphabet, 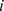 is an element of 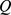 called the initial state, 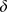 is a partial function from 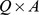 into 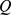 called the transition function, and 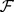 is a subset of 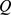 called the set of final states. A finite deterministic Büchi automaton is generally represented as a directed labelled graph whose nodes and labelled edges correspond to the states and transitions of the automaton, respectively.

Given some finite deterministic Büchi automaton 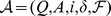, every triple 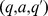 such that 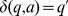 is called a *transition* of 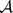. Then, a *path* in 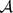 is a sequence of consecutive transitions 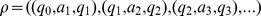, also denoted by 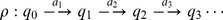. The path 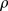 is said to successively *visit* the states 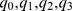 and the word 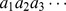 is the *label* of 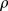. The state 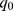 is called the *origin* of path 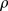 and 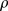 is said to be *initial* if its starting state is initial, i.e. if 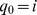. If 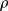 is an infinite path, the set of states visited infinitely many times by 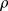 is denoted by 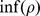.

An infinite initial path 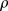 of 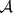 is said to be *successful* if it visits at least one of the final states infinitely often, i.e. if 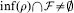. An infinite word is then said to be *recognised* by 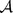 if it is the label of a successful infinite path in 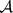. The *language recognised by* 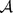, denoted by 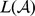, is the set of all infinite words recognised by 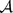.

A *cycle* in 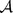 consists of a finite set of states 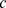 such that there exists a finite path in 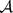 with same initial and final state and visiting precisely all states of 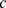. A cycle 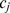 is said to be *accessible* from cycle 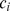 if there exists a path from some state of 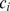 to some state of 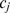. Furthermore, a cycle is called *successful* if it contains a state belonging to 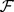, and *non-successful* otherwise.

An *alternating chain of length* 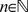 (respectively *co-alternating chain of length* 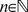) is a finite sequence of 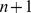 distinct cycles 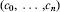 such that 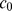 is successful (resp. 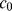 is non-successful), 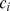 is successful iff 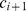 is non-successful, 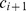 is accessible from 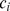, and 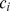 is not accessible from 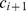, for all 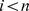. An *alternating chain of length* 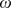 is a sequence of two cycles 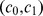 such that 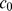 is successful, 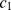 is non-successful, 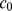 is accessible from 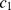, and 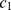 is also accessible from 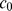 (we recall that 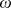 denotes the least infinite ordinal). In this case, cycles 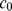 and 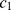 are said to communicate. For any 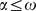, an alternating chain of length 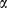 is said to be *maximal* in 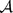 if there is no alternating chain and no co-alternating chain in 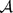 with a length strictly larger than 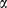. A co-alternating chain of length 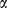 is said to be maximal in 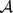 if exactly the same condition holds.

The above definitions are illustrated by the Example S1 and Figure S1 in File S1.

#### Muller Automata

A *finite deterministic Muller automaton* is a 5-tuple 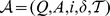, where 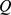, 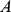, 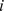, and 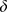 are defined exactly like for deterministic Büchi automata, and 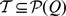 is a set of states' sets called the *table of the automaton*. The notions of transition and path are defined as for deterministic Büchi automata. An infinite initial path 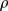 of 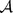 is now called *successful* if 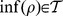. Given a finite deterministic Muller automaton 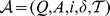, a cycle in 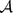 is called *successful* if it belongs to 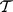, and *non-succesful* otherwise. An infinite word is then said to be *recognised* by 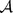 if it is the label of a successful infinite path in 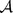, and the 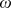 *-language recognised by* 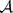, denoted by 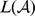, is defined as the set of all infinite words recognised by 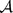. The class of all 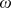-languages recognisable by some deterministic Muller automata is precisely the class of 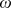 *-rational languages* [79].

It can be shown that deterministic Muller automata are strictly more powerful than deterministic Büchi automata, but have an equivalent expressive power as non-deterministic Büchi automata, Rabin automata, Street automata, parity automata, and non-deterministic Muller automata [81].

**Figure 2:**
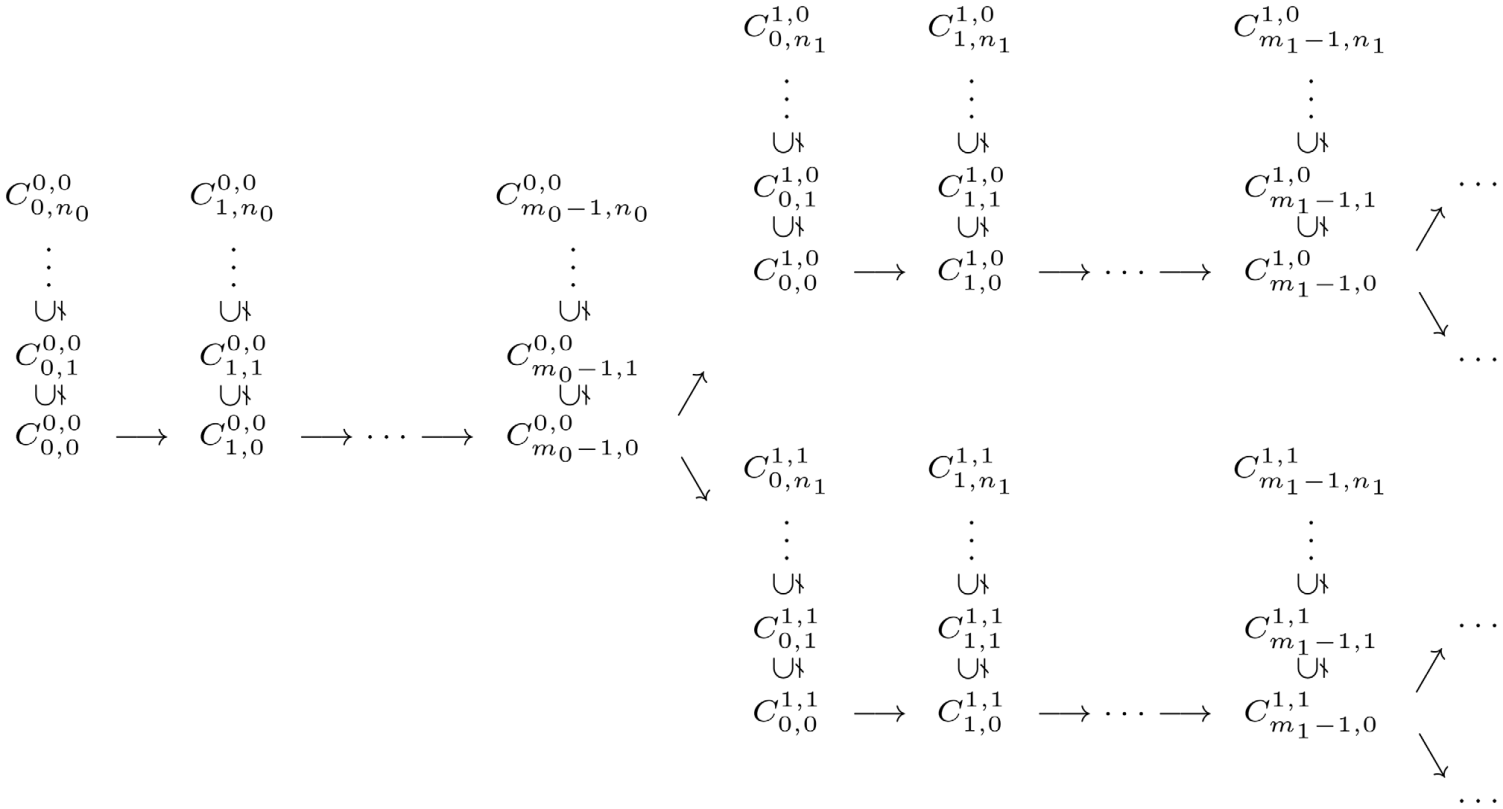
An alternating tree of length 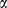, for some ordinal 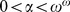. Illustration of the inclusion and accessibility relations between cycles forming an alternating tree of length 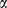.

For each ordinal 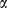 such that 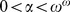, we introduce the concept of an *alternating tree* of length 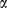 in a deterministic Muller automaton 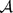, which consists of a tree-like disposition of the successful and non-successful cycles of 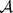 induced by the ordinal 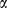, as illustrated in Figure 2. In order to describe this tree-like disposition, we first recall that any ordinal 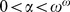 can uniquely be written of the form 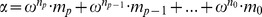, for some 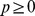, 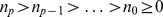, and 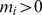. Then, given some deterministic Muller automata 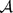 and some strictly positive ordinal 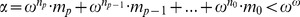, an *alternating tree* (respectively *co-alternating tree*) of length 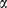 is a sequence of cycles of 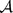 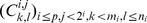 such that:

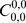
 is successful (respectively non-successful);

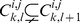
, and 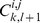 is successful iff 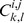 is non-successful;

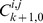
 is accessible from 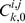, and 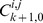 is successful iff 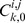 is non-successful;

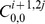
 and 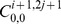 are both accessible from 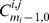, and each 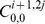 is successful whereas each 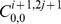 is non-successful.

An alternating tree of length 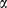 is said to be maximal in 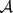 if there is no alternating or co-altenrating tree in 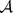 of length 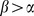. A co-alternating tree of length 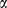 is said to be maximal in 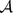 if exactly the same condition holds. An alternating tree of length 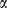 is illustrated in Figure 2.

The above definitions are illustrated by the Example S2 and Figure S2 in File S2.

## Results

### Hierarchical Classification of Neural Networks

Our notion of an attractor refers to a set of states such that the behaviour of the network could forever be confined into that set of states. In other words, an attractor corresponds to a cyclic behaviour of the network produced by an infinite input stream. According to these considerations, we provide a generalisation to this precise infinite input stream context of the classical equivalence result between Boolean neural networks and finite state automata [1]–[3]. More precisely, we show that, under some natural specific conditions on the specification of the type of their attractors, Boolean recurrent neural networks express the very same expressive power as deterministic Büchi automata. This equivalence result enables us to establish a hierarchical classification of neural networks by translating the Wagner classification theory from the Büchi automaton to the neural network context [35]. The obtained classification is intimately related to the attractive properties of the neural networks, and hence provides a new refined measurement of the computational power of Boolean neural networks in terms of their attractive behaviours.

#### Boolean Recurrent Neural Networks and Büchi Automata

We now prove that, under some natural conditions, Boolean recurrent neural networks are computationally equivalent to deterministic Büchi automata. Towards this purpose, we consider that the neural networks include selected elements belonging to an output layer. The activation of the output layer communicates the output of the system to the environment.

Formally, let us consider a recurrent neural network 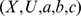, as described in Definition 1, with 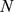 activation cells and 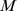 input units. In addition, let us assume that 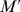 cells chosen among the 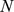 activation cells form the *output layer* of the neural network, denoted by 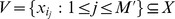. For graphical purpose, the activation cells of the output layer are represented as double-circled nodes in the next figures. Thus, a recurrent neural network is now defined by a tuple 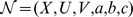. Let us assume also that the specification type of the attractors of a network 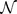 is naturally related to its output layer as follows: an attractor 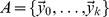 of 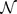 is considered *meaningful* if it contains at least one state where some output cell is spiking, i.e. if there exist 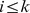 and 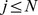 such that 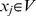 and 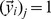; the attractor 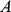 is considered *spurious* otherwise. According to these assumptions, meaningful attractors refer to the cyclic behaviours of the network that induce some response activity of the system via its output layer, whereas spurious attractors refer to the cyclic behaviours of the system that do not evoke any response at all of the output layer.

It can be stated that the expressive powers of Boolean recurrent neural networks and deterministic Büchi automaton are equivalent. As a first step towards this result, the following proposition shows that any Boolean recurrent neural network can be simulated by some deterministic Büchi automaton.

**Proposition 1.** *Let* 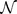 *be some Boolean recurrent neural network provided with an output layer. Then there exists a deterministic Büchi automaton* 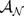 *such that* 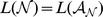 *.*

*Proof.* Let 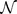 be some neural network given by the tuple 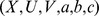, with 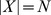, 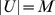, and 
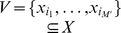
. Consider the deterministic Büchi automaton 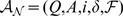, where 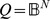, 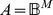, 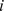 is the 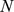-dimensional zero vector, 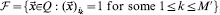, and 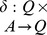 is the function defined by 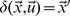 iff 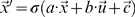. Note that the complexity of the transformation is exponential, since 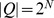 and 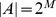.

According to this construction, any infinite evolution 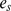 of 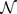 naturally induces a corresponding infinite initial path 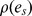 in 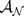. Moreover, by the definitions of meaningful and spurious attractors of 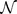, an infinite input stream 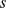 is meaningful for 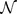 iff 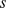 is recognised by 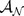. In other words, 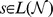 iff 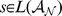, and therefore 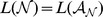.

According to the construction given in the proof of Proposition 1, any infinite evolution of the network 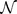 is naturally associated with a corresponding infinite initial path in the automaton 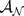, and conversely, any infinite initial path in 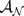 corresponds to some possible infinite evolution of 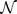. Consequently, there is a biunivocal correspondence between the *attractors* of the network 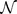 and the *cycles* in the graph of the corresponding Büchi automaton 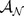. As a result, a procedure to compute all possible attractors of a given network 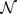 is obtained by firstly constructing the corresponding deterministic Büchi automaton 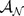 and secondly listing all cycles in the graph of 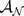.

As a second step towards the equivalence result, we prove now that any deterministic Büchi automaton can be simulated by some Boolean recurrent neural network.

**Proposition 2.** *Let* 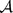 *be some deterministic Büchi automaton over the alphabet* 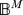 *, with* 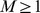 *. Then there exists a Boolean recurrent neural network* 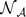 *provided with an output layer such that* 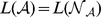 *.*

*Proof.* Let 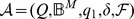 be some deterministic Büchi automaton over alphabet 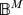, with 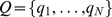, and 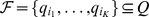. Consider the network 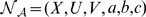 with 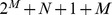 cells given as follows: firstly, 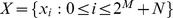, where 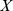 is decomposed into a set of 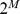 “letter cells” 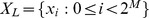, a “delay-cell” 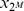, and a set of 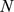 “state cells” 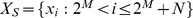; secondly, the set of 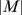 “input units” 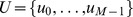, and thirdly, the outptut layer 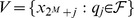. The idea of the simulation is that the “letter cells” and “state cells” of the network 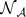 simulate the letters and states currently read and entered by the automaton 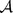, respectively.

**Figure 3:**
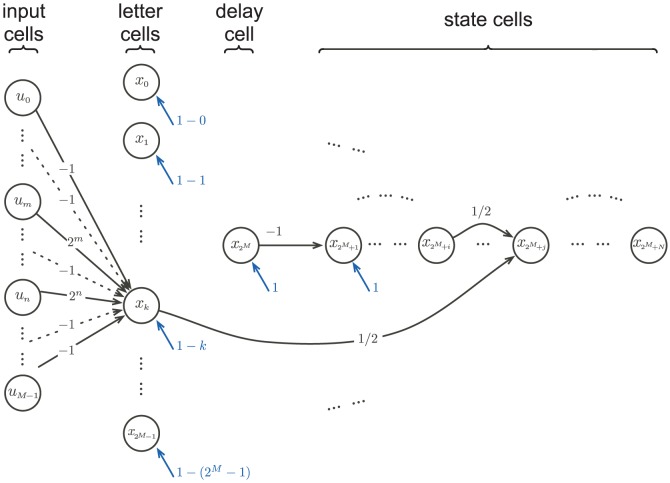
The network 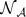 described in the proof of Proposition 2. The network is characterised by a set of 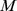 input cells 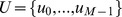 reading the alphabet 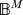, 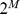 “letter cells” 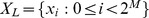, a “delay-cell” 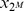, and a set of 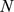 “state cells” 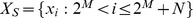. The idea of the simulation is that the “letter cells” and “state cells” of the network 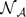 simulate the letters and states currently read and entered by the automaton 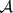, respectively. In this illustration, we assume that the binary decomposition of 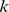 is given by 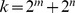, so that the “letter cell” 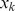 receives synaptic connections of intensities 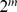 and 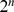 from input cells 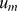 and 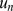, respectively, and it receives synaptic connections of intensities 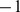 from any other input cells. Consequently, the “letter cell” 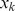 becomes active at time 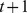 iff the sole input cells 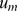 and 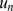 are active at time 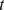. The synaptic connections to other “letter cells” are not illustrated. Moreover, the synaptic connections 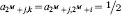 model the transition 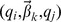 of automaton 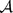. The synaptic connections modelling other transitions are not illustrated.

Towards this purpose, the weight matrices 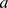, 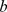, and 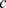 are described as follows. Concerning the matrix 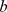: for any 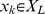, we consider the binary decomposition of 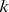, namely 
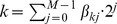
, with 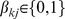, and for any 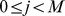, we set the weight 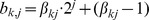; for all other 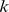, we set 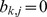, for any 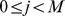. Concerning the matrix 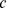: for any 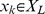, we set 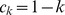; we also set 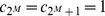; for all other 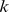, we set 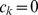. Concerning the matrix 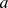: we set 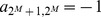, and for any 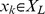 and any 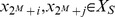, we set 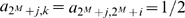 iff 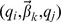 is a transition of 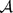; otherwise, for any pair of indices 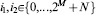 such that 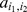 has not been set to 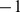 or 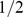, we set 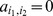. This construction is illustrated in Figure 3.

According to this construction, if we let 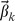 denote the boolean vector whose components are the 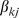
's (for 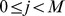), one has that the “letter cell” 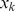 will spike at time 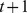 iff the input vector 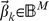 is received at time 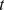. Moreover, at every time step 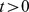, a unique “letter cell” 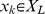 and “state cell” 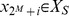 are spiking, and, if 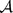 performs the transition 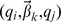 at time 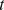, then network 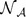 evokes the spiking pattern 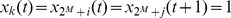. The relation between the final states 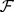 of 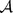 and the output layer 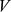 of 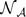 ensures that any infinite input stream 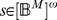 is recognised by 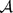 if and only if 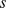 is meaningful for 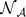. Therefore, 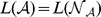.

The proof of Proposition 2 can be generalised to any network dynamics driven by unate local transition functions 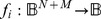, for 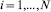, rather than by the 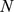 threshold local transition functions defined by Equation 1. Since unate functions are a generalisation of threshold functions, this proof can be interesting in the broader context of switching theory.

Propositions 1 and 2 yield to the following equivalence between recurrent neural networks and deterministic Büchi automata.

**Theorem 1.** *Let* 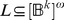 *for some* 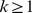 *. Then* 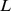 *is recognisable by some Boolean recurrent neural network provided with an output layer iff* 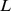 *is recognisable by some deterministic Büchi automaton.*

*Proof.* Proposition 1 shows that every language recognisable by some Boolean recurrent neural network is also recognisable by some deterministic Büchi automaton. Conversely, Proposition 2 shows that every language recognisable by some deterministic Büchi automaton is also recognisable by some Boolean recurrent neural network.

The two procedures given in the proofs of propositions 1 and 2 are illustrated by the Example S3 and Figure S3 in File S3.

#### RNN Hierarchy

In the theory of infinite word reading machines, abstract devices are commonly classified according to the topological complexity of their underlying 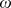-language (i.e., the languages of infinite words that they recognise). Such classifications provide an interesting measurement of the expressive power of various kinds of infinite word reading machines. In this context, the most refined hierarchical classification of 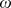-automata – or equivalently, of 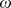-rational languages – is the so-called *Wagner hierarchy* [35].

Here, this classification approach is translated from the 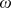-automaton to the neural network context. More precisely, according to the equivalence given by Theorem 1, the Wagner hierarchy can naturally be translated from Büchi automata to Boolean neural networks. As a result, a hierarchical classification of first-order Boolean recurrent neural networks is obtained. Interestingly, the obtained classification is tightly related to the attractive properties of the networks, and, more precisely, refers to the ability of the networks to switch between meaningful and spurious attractive behaviours along their evolutions. Hence, the obtained hierarchical classification provides a new measurement of complexity of neural networks associated with their abilities to switch between different types of attractors along their evolutions.

As a first step, the following facts and definitions need to be introduced. To begin with, for any 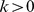, the space 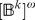 can naturally be equipped with the product topology of the discrete topology over 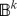. Accordingly, one can show that the basic open sets of 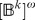 are the sets of infinite sequences of 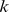-dimensional Boolean vectors which all begin with a same prefix, or formally, the sets of the form 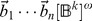, where 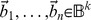. An open set is then defined as a union of basic open sets. Moreover, as usual, a function 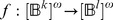 is said to be continuous iff the inverse image by 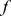 of every open set of 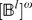 is an open set of 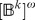. Now, given two Boolean recurrent neural networks 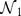 and 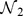 with 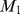 and 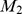 input units respectively, we say that 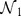 *reduces* (or *Wadge reduces* or *continuously reduces*) to 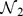, denoted by 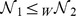, iff there exists a continuous function 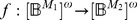 such that, for any input stream 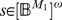, one has 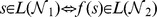, or equivalently, such that 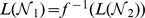 [78]. Intuitively, 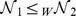 iff the problem of determining whether some input stream 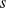 belongs to the neural language of 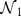 (i.e. whether 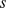 is meaningful for 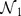) reduces via some simple function 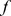 to the problem of knowing whether 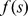 belongs to the neural language of 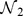 (i.e. whether 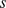 is meaningful for 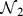). The corresponding strict reduction, equivalence relation, and incomparability relation are then naturally defined by 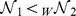 iff 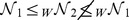, as well as 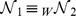 iff 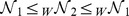, and 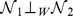 iff 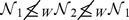. Moreover, a network 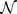 is called *self-dual* if 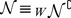; it is called *non-self-dual* if 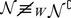, which can be proved to be equivalent to saying that 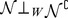 [78]. We recall that the network 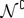, as defined in Section “Formal Definitions”, corresponds to the network 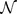 whose type specification of its attractors has been inverted. Consequently, 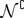 does not correspond *a priori* to some neural network provided with an output layer. By extension, an 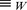-equivalence class of networks is called *self-dual* if all its elements are self-dual, and *non-self-dual* if all its elements are non-self-dual.

The continuous reduction relation over the class of Boolean recurrent neural networks naturally induces a hierarchical classification of networks formally defined as follows:

**Definition 3.** The collection of all Boolean recurrent neural networks ordered by the reduction “
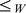
” is called the RNN *hierarchy*.

We now provide a precise description of the RNN hierarchy. The result is obtained by drawing a parallel between the RNN hierarchy and the restriction of the Wagner hierarchy to Büchi automata. For this purpose, let us define the *DBA hierarchy* to be the collection of all deterministic Büchi automata over multidimensional Boolean alphabets 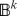 ordered by the continuous reduction relation “
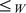
”. More precisely, given two deterministic Büchi automata 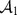 and 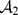, we set 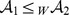 iff there exists a continuous function 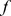 such that, for any input stream 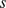, one has 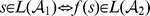. The following result shows that the RNN hierarchy and the DBA hierarchy are actually isomorphic. Moreover, a possible isomorphism is given by the mapping described in Proposition 1 which associates to every network 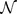 a corresponding deterministic Büchi automaton 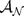.

**Proposition 3.** *The RNN hierarchy and the DBA hierarchy are isomorphic.*

*Proof.* Consider the mapping described in Proposition 1 which associates to every network 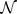 a corresponding deterministic automaton 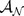. We prove that this mapping is an embedding from the RNN hierarchy into the DBA hierarchy. Let 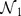 and 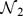 be any two networks, and let 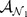 and 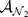 be their corresponding deterministic Büchi automata. Proposition 1 ensures that 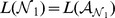 and 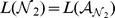. Hence, one has 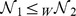 iff by definition there exists a continuous function 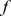 such that 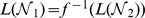 iff there exists a continuous function 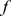 such that 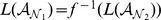, iff by definition 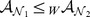. Therefore 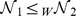 iff 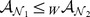. It follows that 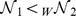 iff 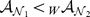, proving that the considered mapping is an embedding. We now show that, up to the continuous equivalence relation “
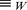
”, this mapping is also onto. Let 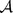 be some deterministic Büchi automaton. By Proposition 2, there exists a network 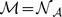 such that 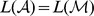. Moreover, by Proposition 1, the automaton 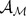 satisfies 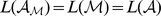. It follows that for any infinite input stream 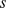, one has 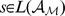 iff 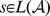, meaning that both 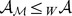 and 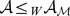 hold, and thus 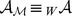. Therefore, for any deterministic Büchi automaton 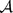, there exists a neural network 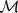 such that 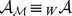, meaning precisely that up to the continuous equivalence relation “
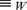
”, the mapping 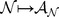 described in Proposition 1 is onto. This concludes the proof.

**Figure 4:**
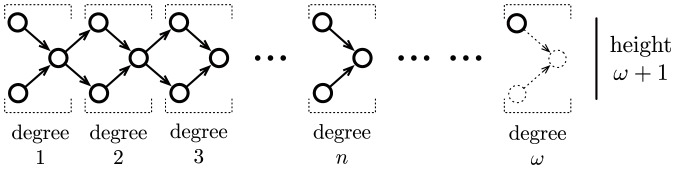
The RNN hierarchy. An infinite alternating succession of pairs of non-self-dual classes of networks followed by single self-dual classes of networks, all of them overhung by an additional single non-self-dual class at the first limit level. Circles represent the equivalence classes of networks (with respect to the relation “
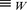
”) and arrows between circles represent the strict reduction “
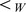
” between all elements of the corresponding classes.

By Proposition 3 and the usual results of the DBA hierarchy, a precise description of the RNN hierarchy can be given. First of all, the RNN hierarchy is well-founded, i.e. there is no infinite strictly descending sequence of networks 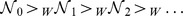. Moreover, the maximal strict chains in the RNN hierarchy have length 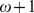, meaning that the RNN hierarchy has a height of 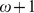. (A strict chain of length 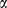 in the RNN hierarchy is a sequence of neural networks 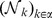 such that 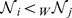 iff 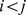; a strict chain is said to be maximal if its length is at least as large as the length of every other strict chain.) Furthermore, the maximal antichains of the RNN hierarchy have length 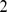, meaning that the RNN hierarchy has a width of 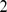. (An antichain of length 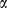 in the RNN hierarchy is a sequence of neural networks 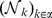 such that 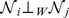 for all 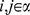 with 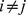; an antichain is said to be maximal if its length is at least as large as the length of every other antichain.) More precisely, it can be shown that incomparable networks are equivalent (for the relation 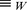) up to complementation, i.e., for any two networks 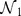 and 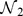, one has 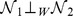 iff 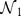 and 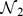 are non-self-dual and 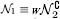. These properties imply that, up to equivalence and complementation, the RNN hierarchy is actually a well-ordering. In fact, the RNN hierarchy consists of an infinite alternating succession of pairs of non-self-dual and single self-dual classes, overhung by an additional single non-self-dual class at the first limit level 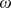, as illustrated in Figure 4.

For convenience reasons, the degree of a network 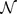 in the RNN hierarchy is defined such that the same degree is shared by both non-self-dual networks at some level and self-dual networks located just one level higher, as illustrated in Figure 4:
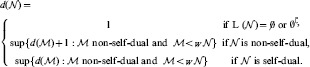
Moreover, the equivalence between the DBA and RNN hierarchies ensures that the RNN hierarchy is actually decidable, in the sense that there exists an algorithmic procedure which is able to compute the degree of any network in the RNN hierarchy. All the above properties of the RNN hierarchy are summarised in the following result.

**Theorem 2.** *The RNN hierarchy is a decidable pre-well-ordering of width* 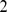 *and height* 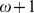 *.*

*Proof.* The DBA hierarchy consists of a decidable pre-well-ordering of width 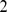 and height 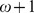 [79]. Proposition 3 ensures that the RNN hierarchy and the DBA hierarchy are isomorphic.

The following result provides a detailed description of the decidability procedure of the RNN hierarchy. More precisely, it is shown that the degree of a network 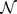 in the RNN hierarchy corresponds precisely to the maximal number of times that this network might switch between visits of meaningful and spurious attractors along some evolution.

**Theorem 3.** *Let* 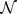 *be some network provided with an additional specification of an output layer,* 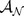 *be the corresponding deterministic Büchi automaton of* 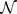 *, and* 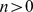 *.*

- *If there exists in* 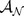 *a maximal alternating chain of length* 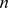 *and no maximal co-alternating chain of length* 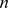 *, then* 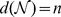 *and* 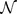 *is non-self-dual.*
- *Symmetrically, if there exists in* 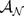 *a maximal co-alternating chain of length* 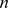 *but no maximal alternating chain of length* 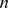 *, then also* 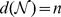 *and* 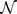 *is non-self-dual.*
- *If there exist in* 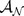 *a maximal alternating chain of length* 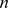 *as well as a maximal co-alternating chain of length* 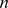 *, then* 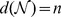 *and* 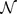 *is self-dual.*
- *If there exist in* 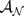 *a maximal alternating chain of length* 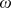 *, then* 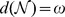 *and* 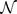 *is non-self-dual.*

*Proof.* By Proposition 3, the degree of a network 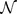 in the RNN hierarchy is equal to the degree of its corresponding deterministic Büchi automaton 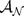 in the DBA hierarchy. Moreover, the degree of a deterministic Büchi automaton in the DBA hierarchy corresponds precisely to the length of a maximal alternating or co-alternating chain contained in this automaton [79].

By Theorem 3, the decidability procedure of the degree of a neural network 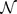 in the RNN hierarchy consists firstly in translating the network 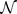 into its corresponding deterministic Büchi automaton 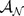, as described in Proposition 1, and secondly in returning the ordinal 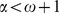 corresponding to the length of a maximal alternating chain or co-alternating chain contained in 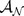. Note that this procedure can clearly be achieved by some graph analysis of the automaton 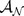, since the graph of 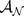 is always finite. Furthermore, since alternating and co-alternating chains are defined in terms of cycles in the graph of the automaton, then according to the biunivocal correspondence between cycles in 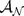 and attractors of 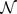, it can be deduced that the complexity of a network in the RNN hierarchy is in fact directly related to the attractive properties of this network.

More precisely, it can be observed that the complexity measurement provided by the RNN hierarchy actually corresponds to the maximal number of times that a network might alternate between visits of meaningful and spurious attractors along some evolution. Indeed, the existence of a maximal alternating or co-alternating chain 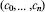 of length 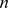 in 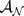 means that every infinite initial path in 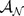 might alternate at most 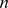 times between visits of successful and non-successful cycles. Yet this is precisely equivalent to saying that every evolution of 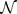 can only alternate at most 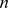 times between visits of meaningful and spurious attractors before eventually becoming trapped forever by a last attractor. In this case, Theorem 3 ensures that the degree of 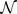 is equal to 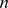. Moreover, the existence of an alternating chain 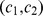 of length 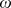 in 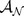 is equivalent to the existence of an infinite initial path in 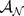 that might alternate infinitely many times between visits of the cycles 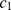 and 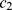. This is equivalent to saying that there exists an evolution of 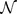 that might alternate infinitely many times between visits of a meaningful and a spurious attractor. By Theorem 3, the degree of 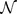 is equal to 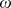 is this case. Therefore, the RNN hierarchy provides a new measurement of complexity of neural networks according to their ability to maximally alternate between different types of attractors along their evolutions.

Finally, the decidability procedure of the RNN hierarchy is illustrated by the Example S4 in File S4.

### Refined Hierarchical Classification of Neural Networks

In this section, we show that by relaxing the restrictions on the specification of the type of their attractors, the networks significantly increase their expressive power from deterministic Büchi automata up to Muller automata [80]. Hence, by translating once again the Wagner classification theory from the Muller automaton to the neural network context, another more refined hierarchical classification of Boolean neural networks can be obtained. The obtained classification is also tightly related to the attractive properties of the networks, and hence provides once again a new refined measurement of complexity of Boolean recurrent neural networks in terms of their attractive behaviours.

#### Boolean Recurrent Neural Networks and Muller Automata

The assumption that the networks are provided with an additional description of an output layer, which would subsequently influence the type specifications (meaningful/spurious) of their attractors, is not necessary anymore from this point onwards. Instead, let us assume that, for any network, the precise classification of its attractors into meaningful and spurious types is known. How the meaningfulness of the attractors is determined is an issue that is not considered here. For instance, the specification of the type of each attractor might have been determined by its neurophysiological significance with respect to measurable observations associated to certain behaviours or sensory discriminations. Formally, in this section, a recurrent neural network consists of a tuple 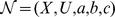, as described in Definition 1, but also provided with an additional specification into meaningful and spurious type for each one of its attractors.

We now prove that, by totally relaxing the restrictions on the specification of the type of their attractors, the Boolean neural networks significantly increase their expressive powers from deterministic Büchi automata up to Muller automata. The following straightforward generalisation of Proposition 1 states that any such Boolean recurrent neural network can be simulated by some deterministic Muller automaton.

**Proposition 4.** *Let* 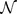 *be some Boolean recurrent neural network provided with a type specification of each of its attractors. Then there exists a deterministic Muller automaton* 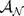 *such that* 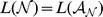 *.*

*Proof.* Let 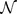 be given by the tuple 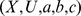, with 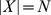, 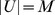, and let the meaningful attractors of 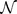 be given by 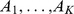, all others being spurious. Now, consider the deterministic Muller automaton 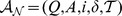, where 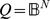, 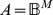, 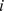 is the 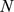-dimensional zero vector, 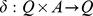 is defined by 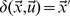 iff 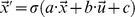, and 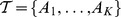. According to this construction, any input stream 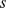 is meaningful for 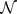 iff 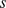 is recognised by 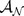. In other words, 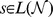 iff 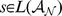, and therefore 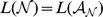.

Conversely, as a generalisation of Proposition 2, we can prove that any deterministic Muller automaton can be simulated by some Boolean recurrent neural network provided with a suitable type specification of its attractors.

**Proposition 5.** *Let* 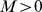 *and let* 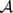 *be some deterministic Muller automaton over the alphabet* 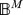 *. Then there exists a Boolean recurrent neural network* 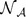 *provided with a type specification of each of its attractors such that* 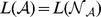 *.*

*Proof.* Let 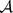 be given by the tuple 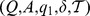, with 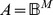, 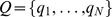 and 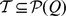. Now, consider the network 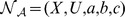 described in the proof of Proposition 2. It remains to define which are the meaningful and spurious attractors of 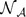. As mentioned in the proof of Proposition 2, at every time step 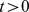, only one among the “state cells” 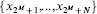 is spiking. Hence, for any state 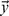 of 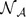 that might occur at some time step 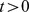, let 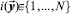 be the index such that 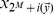 is the unique “state cell” which is spiking during state 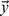. An attractor 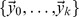 of 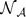 is then said to be meaningful iff 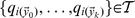.

Consequently, for any infinite infinite sequence 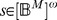, the infinite path 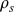 in 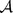 satisfies 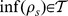 iff the evolution 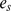 in 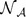 is such that 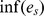 is a meaningful attractor. Therefore, 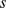 is recognised by 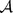 iff 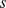 is meaningful for 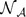, showing that 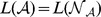.

Propositions 4 and 5 yield the following equivalence between Boolean recurrent neural networks and deterministic Muller automata.

**Theorem 4.** *Let* 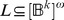 *for some* 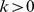 *. Then the following conditions are equivalent:*

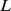
 *is recognisable by some Boolean recurrent neural network provided with a type specification of its attractors;*

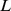
 *is recognisable by some deterministic Muller automaton;*

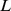
 *is* 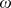 *-rational.*

*Proof.* The equivalence between conditions a and b is given by propositions 4 and 5. The equivalence between conditions b and c is a well-known result of automata theory [79].

The two procedures described in the proofs of propositions 4 and 5 are illustrated by the Example S5 and Figure S4 in File S5.

#### Complete RNN Hierarchy

In this section, we prove that the collection of Boolean recurrent neural networks ordered by the continuous reduction corresponds to a refined hierarchical classification of height 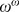. This classification is directly related to the attractive properties of the networks, and therefore provides a new refined measurement of complexity of neural networks according to their attractive behaviours. This hierarchical classification is formally defined as follows.

**Definition 4.** *The collection of all Boolean recurrent neural networks provided with a type specification of their attractors ordered by the continuous reduction “* 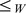 *” is called the complete RNN hierarchy.*

Like in Section “RNN Hierarchy”, a precise characterisation of the complete RNN hierarchy can be obtained by translating the Wagner classification theory from the Muller automaton to the neural network context. For this purpose, we shall consider the collection of all deterministic Muller automata over multidimensional Boolean alphabets 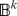 ordered by the continuous reduction “
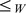
”. This hierarchy is commonly referred to as the *Wagner hierarchy* [35]. A generalisation of Proposition 3 shows that the complete RNN hierarchy and the Wagner hierarchy are isomorphic, and a possible isomorphism is also given by the mapping described in Proposition 4 which associates to every network 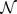 a corresponding deterministic Muller automaton 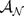.

**Proposition 6.** *The complete RNN hierarchy and the Wagner hierarchy are isomorphic.*

*Proof.* Consider the mapping described in Proposition 4 which associates to every network 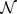 a corresponding deterministic Muller automaton 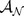. A similar reasoning as the one presented in the proof of Proposition 3 shows that this mapping is an isomorphism between the complete RNN hierarchy and the Wagner hierarchy.

**Figure 5:**
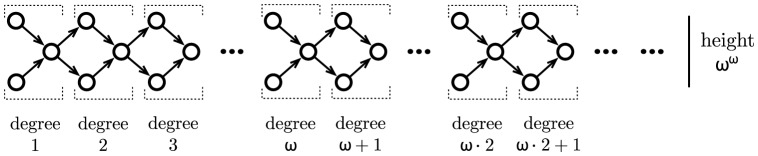
The complete RNN hierarchy. A transfinite alternating succession of pairs of non-self-dual classes of networks followed by single self-dual classes of networks, with non-self-dual classes at each limit level.

By Proposition 6 and the usual results on the Wagner hierarchy [35], the following precise description of the complete RNN hierarchy can be given. First of all, like the RNN hierarchy, the complete RNN hierarchy also consists of a pre-well ordering of width 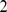, and any two networks 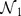 and 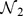 satisfy the incomparability relation 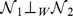 iff 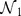 and 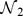 are non-self-dual networks such that 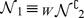. However, while the RNN hierarchy has only height 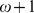, the complete RNN hierarchy shows a height of 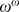 levels. In fact, the complete RNN hierarchy consists of an infinite alternating succession of pairs of non-self-dual and single self-dual classes, with non-self-dual classes at each limit level, as illustrated in Figure 5. For convenience reasons, the degree 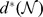 of a network 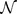 in the complete RNN hierarchy is also defined such that the same degree is shared by both non-self-dual networks at some level and self-dual networks located just one level higher, namely:
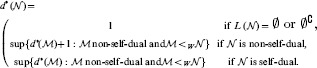
Besides, the isomorphism between the Wagner hierarchy and the complete RNN hierarchy ensures that the complete RNN hierarchy is actually decidable, in the sense that there exists an algorithmic procedure allowing to compute the degree of any network in the complete RNN hierarchy. The following theorem summarises the properties of the complete RNN hierarchy.

**Theorem 5.** *The complete RNN hierarchy is a decidable pre-well-ordering of width* 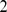 *and height* 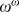 *.*

*Proof.* The Wagner hierarchy consists of a decidable pre-well-ordering of width 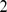 and height 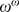 [35]. Proposition 6 ensures that the complete RNN hierarchy and the Wagner hierarchy are isomorphic.

The following result provides a detailed description of the decidability procedure of the complete RNN hierarchy. More precisely, it is shown that the degree of a network 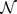 in the RNN hierarchy corresponds precisely to the largest ordinal 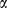 such that there exists an alternating tree or a co-alternating tree of length 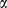 in the deterministic Muller automaton 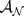.

**Theorem 6.** *Let* 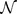 *be some Boolean recurrent network provided with a type specification of its attractors,* 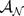 *be the corresponding deterministic Muller automaton of* 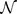 *, and* 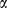 *be an ordinal such that* 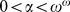 *.*

- *If there exists in* 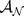 *a maximal alternating tree of length* 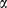 *and no maximal co-alternating tree of length* 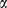 *, then* 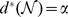 *and* 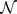 *is non-self-dual.*
- *If there exists in* 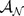 *a maximal co-alternating tree of length* 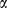 *and no maximal alternating tree of length* 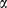 *, then* 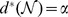 *and* 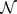 *is non-self-dual.*
- *If there exist in* 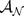 *both a maximal alternating tree as well as a maximal co-alternating tree of length* 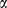 *, then* 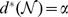 *and* 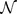 *is self-dual.*

*Proof.* By Proposition 6, the degree of a network 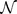 in the complete RNN hierarchy is equal to the degree of its corresponding deterministic Muller automaton 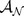 in the Wagner hierarchy. Moreover, the degree of a deterministic Muller automaton in the Wagner hierarchy corresponds precisely to the length of a maximal alternating or co-alternating tree contained in this automaton [35], [82].

The decidability procedure of the degree of a neural network 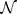 in the complete RNN hierarchy thus consists in first translating the network 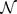 into its corresponding deterministic Muller automaton 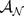, as described in Proposition 4, and then returning the ordinal 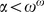 corresponding to the length of a maximal alternating tree, or co-alternating tree, contained in 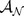. Note that this procedure can be achieved by some graph analysis of the automaton 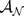, since the graph of 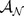 is always finite.

By Theorem 6, the degree of a neural network 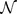 in the complete RNN hierarchy corresponds precisely to the length of a maximal alternating, or co-alternating, tree contained in 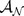. Since alternating and co-alternating trees are defined in terms of cycles in the graph of the Muller automaton, and according to the biunivocal correspondence between cycles in 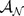 and attractors of 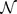, it can be deduced that, like for the RNN hierarchy, the complexity of a network in the complete RNN hierarchy is also directly related to the attractive properties of this network. In fact, the complexity measurement provided by the complete RNN hierarchy refers to the maximal number of times that a network might alternate between visits of meaningful and spurious attractors along some evolution.

More precisely, the 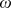 first levels of the complete RNN hierarchy provide a classification of the collection of networks that might switch at most *finitely* many times between different types of attractors along their evolutions. Indeed, by Theorem 6, for any 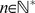, a network 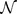 satisfies 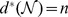 iff 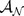 contains a maximal alternating, or co-alternating, tree of length 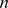. In other words, for any 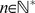, a network 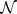 satisfies 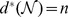 iff 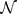 is able to switch at most 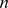 times between visits of different types of attractors during all its possible evolutions.

The levels of transfinite degrees provide a refined classification of the collection of networks that are able to alternate *infinitely* many times between different types of attractors. Indeed, according to Theorem 6, for any ordinal 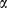 such that 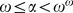, a network 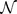 satisfies 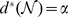 iff 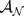 contains a maximal alternating or co-alternating tree of length 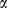. Since 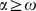, this implies that the graph of 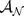 necessarily contains at least two cycles 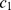 and 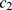 such that 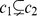 and 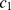 is successful iff 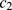 is non-successful. But since 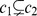, it follows that 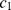 and 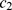 are both accessible one from the other in the graph of 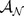. By the biunivocal correspondence between cycles and attractors, the network 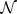 contains at least the two attractors 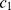 and 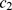, and the accessibility between those ensures that the network is capable of alternating infinitely often between visits of 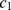 and 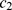 along some evolution. In fact, the collection of levels of transfinite degrees of the complete RNN hierarchy provides a refined classification of these potentially infinitely switching networks based on the intricacy of their underlying attractive structures (tree-like representation induced by the inclusion and accessibility relations between the attractors, as illustrated in Figure 2).

It can be noticed, according to the definition of alternating and co-alternating trees, that if some given Muller automaton contains either an alternating or a co-alternating tree of length 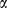 in its underlying graph, then this automaton also necessarily contains in its graph both an alternating and a co-alternating tree of length 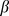, for all 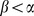. Therefore, any network of the complete RNN hierarchy is capable of disclosing an attractive behaviour analogous to any other network of strictly smaller degree. In this precise sense, a network of the complete RNN hierarchy potentially contains in its structure all the possible attractive behaviours of every other networks of strictly smaller degrees. In this framework, the concept of alternation between different types of attractors corresponds to the transient trajectories between attractor basins, a concept referred to as “itinerancy” elsewhere in the literature [51], [65]–[67], [83], [84].

The decidability procedure of the complete RNN hierarchy is illustrated by the Example S6 in File S6.

It is worth noting that the complete RNN hierarchy can actually be seen as a proper extension of the RNN hierarchy. Indeed, the next result shows that the networks of RNN hierarchy and the networks of the specific initial segment of length 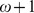 of the complete RNN hierarchy recognise precisely the same languages. In this precise sense, the RNN hierarchy consists of an initial segment of length 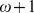 of the complete RNN hierarchy.

**Proposition 7.** *Let* 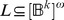 *. Then* 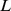 *is recognisable by some network* 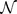 *of the RNN hierarchy iff* 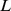 *is also recognisable by some network* 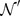 *of the complete RNN hierarchy such that either* 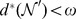 *or* 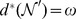 *and* 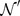 *contains a maximal co-alternating tree of length* 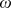 *but no maximal alternating tree of length* 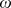 *.*

*Proof.* Given any deterministic Muller automaton 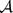, let the degree of 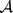 in the Wagner hierarchy be denoted by 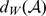. Then, the relationship between the DBA and the Wagner hierarchies ensures that 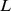 is recognisable by some deterministic Büchi automaton iff 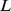 is also recognisable by some deterministic Muller automaton 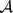 such that either 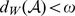 or 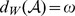 and 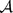 contains a maximal co-alternating tree of length 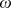 but no maximal alternating tree of length 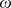 [79]. Theorems 1 and 4 together with Proposition 6 allow to translate these results to the neural network context, and therefore lead to the conclusion.

We recall that the RNN hierarchy consists of the classification of networks whose attractors' type specifications are induced by the existence of an output layer, whereas the complete RNN hierarchy consists of the classification of networks whose attractors' type specifications are *a priori* given without any restriction at all. For any ordinal 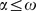, the two notions of alternating chain and alternating tree of length 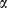 coincide. Hence, by Theorem 3 and Theorem 6, the two decidability procedures of the RNN hierarchy and the complete RNN hierarchy reduce to the very same, and the decidability procedures simply consist in computing the length of a maximal alternating or co-alternating tree contained in the underlying automata.

**Figure 6:**
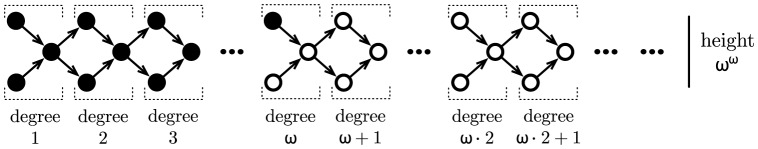
Comparison between the RNN and the complete RNN hierarchies. The RNN hierarchy, depicted by the sequence of blacks classes, consists of an initial segment of length 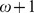 of the complete RNN hierarchy, which has height 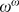.

However, it is important to clarify the difference between the RNN hierarchy and the complete RNN hierarchy, illustrated in Figure 6. The restriction on the type specification of the attractors imposed by the existence of an output layer ensures that the networks of the RNN hierarchy will never be able to contain a maximal alternating or co-alternating tree of length strictly larger than 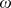 in their underlying Büchi automata. Indeed, if 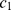 and 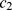 are two cycles in a deterministic Büchi automaton such that 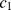 is successful and 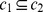, then 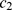 is necessarily also successful (since it visits the same final states as 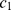 plus potentially some other ones), meaning that no meaningful cycle could ever be included in some spurious cycle in a deterministic Büchi automaton; consequently, the maximal number alternations between different type of cycles that can be found in a deterministic Büchi automaton is bounded by one – a spurious cycle included in a meaningful cycle – and therefore no alternating or co-alternating trees of length strictly larger than 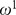 will never exist in a deterministic Büchi automaton. From this observation, it follows that the degree of any network of the RNN hierarchy is bounded by 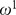, meaning that the length of the RNN hierarchy cannot exceed 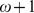, whereas the length of the complete RNN hierarchy climbs up to 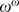, as illustrated by Figure 6.

### The “basal ganglia-thalamocortical network”

#### Neurobiological description

In order to illustrate the application of our method to a case study, we have considered one of the main systems of the brain involved in information processing, the basal ganglia-thalamocortical network. This network is formed by several parallel and segregated circuits involving different areas of the cerebral cortex, striatum, pallidum, thalamus, subthalamic nucleus and midbrain [85]–[94]. This network has been investigated for many years in particular in relation to disorders of the motor system and of the sleep-waking cycle. The simulations were generally performed by considering the basal ganglia-thalamocortical network as a circuit composed of several interconnected areas, each area being modeled by a network of spiking neurons, and were analysed using statistical approaches based on mean-field theory [95]–[106].

In the basal ganglia-thalamocortical network are several types of connections and transmitters. Based on the observation that almost all neurons of the central nervous system can be subdivided into projection neurons and interneurons, we consider the connections mediated by projection neurons, both glutamatergic excitatory projections and GABAergic inhibitory projections, as part of an information transmitting system. The local connections established by the interneurons, i.e. the connections remaining confined within a small distance from the projection neurons, are considered forming part of a regulatory system. The other connections, mainly produced by different types of projection neurons, i.e. the dopaminergic (including those from the substantia nigra pars compacta like the nigrostriatal and those from the ventromedial tegmental area), cholinergic (including those from the basal forebrain), the noradrenergic (including those from locus coeruleus), serotoninergic (including those from the dorsal raphe), histaminergic (from the tuberomamillary nucleus) and orexinergic projections (from the lateral and posterior hypothalamus) are considered forming part of a modulatory system. The three systems, information transmitting, regulatory and modulatory have an extensive pattern of reciprocal interconnectivity at various levels that is not addressed in this paper.

**Figure 7:**
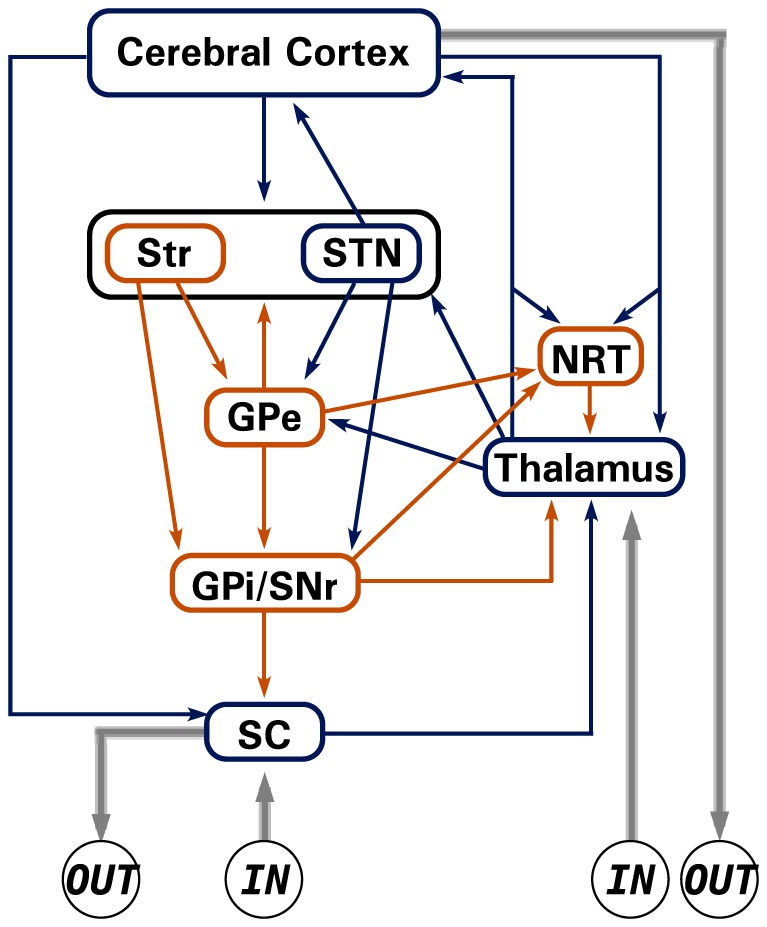
Model of the basal ganglia-thalamocortical network. The network is constituted of 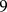 different interconnected brain areas, each one represented by a single node in the Boolean neural network model: superior colliculus (SC), Thalamus, thalamic reticular nucleus (NRT), Cerebral Cortex, the striatopallidal and the striatonigral components of the striatum (Str), the subthalamic nucleus (STN), the external part of the pallidum (GPe), and the output nuclei of the basal ganglia formed by the GABAergic projection neurons of the intermediate part of the pallidum and of the substantia nigra pars reticulata (GPi/SNR). We consider also the inputs (IN) from the ascending sensory pathway and the motor outputs (OUT). The excitatory pathways are labeled in blue and the inhibitory ones in orange.

A characteristic of all the circuits of the basal ganglia-thalamocortical network is a combination of “open” and “closed” loops with ascending sensory afferences reaching the thalamus and the midbrain, and with descending motor efferences from the midbrain (the tectospinal tract) and the cortex (the corticospinal tract). We assume that the encoding of a large amount of the information treated by the brain is performed by recurrent patterns of activity circulating in the information transmitting system. For this reason, we focus our attention on the complexity of the dynamics that may emerge from that system. To this purpose, we present a Boolean recurrent neural network model of the information transmitting system of the basal ganglia-thalamocortical network, illustrated by Figure 7.

The pattern of connectivity corresponds to the wealth of data reported in the literature [85]–[94]. We assume that each brain area is formed by a neural network and that the network of brain areas corresponding to the basal ganglia-thalamocortical network can be modeled by a Boolean neural network formed by 9 nodes: superior colliculus (SC), Thalamus, thalamic reticular nucleus (NRT), Cerebral Cortex, the two functional parts (striatopallidal and the striatonigral components) of the striatum (Str), the subthalamic nucleus (STN), the external part of the pallidum (GPe), and the output nuclei of the basal ganglia formed by the GABAergic projection neurons of the intermediate part of the pallidum and of the substantia nigra pars reticulata (GPi/SNR).

We consider the ascending sensory pathway (IN), that reaches SC and the Thalamus. SC does not send other projections to the system and sends a projection outside of the system (OUT), to the motor system. The thalamus sends excitatory connections within the system via the thalamo-pallidal, thalamo-striatal and thalamo-cortical projections. Notice that STN receives also an excitatory projection from the Thalamus. NRT receives excitatory collateral projections from both the thalamo-cortical and cortico-thalamic projections. In turn, NRT sends an inhibitory projection to the Thalamus. The Cerebral Cortex receives also an excitatory input from STN and sends corticofugal projections to the basal ganglia (striatum and STN), to the thalamus, to the midbrain and to the motor system (OUT). The only excitatory nucleus of the basal ganglia is STN, that sends projections to the Cerebral Cortex, to GPe and to GPi/SNR. In the striatum (Str) the striatopallidal neurons send inhibitory projections to GPe and the striatonigral neurons send inhibitory projections to GPi/SNR, via the so-called “direct” pathway. The pallidum (GPe) plays a paramount role because it is an inhibitory nucleus, with reciprocal connections back to the striatum and to STN and a downstream inhibitory projection to GPi/SNR via the so-called “indirect” pathway. It is interesting to notice the presence of inhibitory projections that inhibit the inhibitory nuclei within the basal ganglia, thus leading to a kind of “facilitation”, but also inhibitory projections that inhibit RTN, that is a major nucleus in regulating the activity of the thalamus. The connectivity of the Boolean model of the basal ganglia-thalamocortical network is described by the adjacency matrix of the network in Table 1.

**Table 1:** The adjancency matrix of the Boolean model of the basal ganglia-thalamocortical network.

Source		Target								
Node	Name	SC	Thalamus	RTN	GPi/SNr	STN	GPe	Str-D2	Str-D1	CCortex
1	SC		1							
2	Thalamus			1		1	1	1	1	1
3	RTN		−1							
4	GPi/SNr	−1	−1	−1						
5	STN				2		2			2
6	GPe			−1/2	−1/2	−1/2		−1/2	−1/2	
7	Str-D2						−1			
8	Str-D1				−1/2		−1/2			
9	Cer. Cortex	1/2	1/2	1/2		1/2		1/2	1/2	

#### Computation of attractor-based complexity

For sake of simplicity, we consider that the two inputs to the basal ganglia-thalamocortical network (Figure 7) are reduced to 1 input node sending projections to Thalamus and SC with synaptic weight equal to 1. We reduce our neurobiological model to a Boolean recurrent neural network 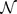 that contains 9 activation nodes and 1 input node. Every activation node can be either active or quiet, which means 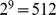 possible states for the network 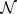. Every state of 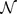 is represented by a 9-dimensional Boolean vector describing the sequence of active and quiet activation nodes. For example, the network state 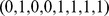 means that the nodes #1 (SC), #3 (RTN) and #4 (GPi/SNR) are quiet, whereas every other activation node is active.

In this section, we provide a practical illustration of our new attractor-based complexity measurement applied to the simplified model of the basal ganglia-thalamocortical network. Since the behaviour of network 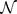 is not determined by any designated output layer, the attractor-based complexity of 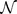 will be measured with respect to the complete RNN hierarchy rather than with respect to the RNN hierarchy, as described in Section “Complete RNN Hierarchy”. According to these considerations, as mentioned in Theorem 6, the attractor-based complexity of network 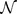 relies on the graphical structure of its corresponding deterministic Muller automaton 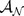. Hence, we shall now describe the structure of the deterministic Muller automaton 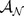 associated to network 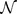.

**Figure 8:**
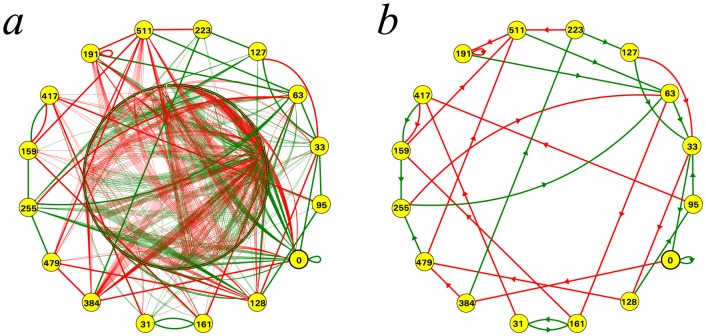
Deterministic Muller automaton based on the “basal ganglia-thalamocortical” network of Figure 7. **a.** The graph of the automaton 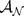 associated to network 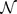 contains 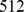 states and 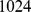 directed transitions. The colours of the transitions represent their labels: green for label 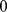 and red for label 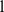. For sake of readability, the directions of the transitions have been removed. The states and transitions of the strongly connected component 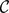 of 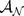 have been pulled out of the central graph and drawn in larger font. **b.** The graph of the strongly connected component 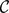 of 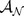. Every state and transition of 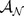 that does not belong to 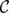 has been erased. The directions of the transitions are indicated by the arrowheads.

Firstly, as mentioned in the proof of Proposition 4, the states of the Muller automaton 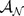 correspond precisely to the states of network 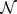. Hence, the deterministic Muller automaton associated to the basal ganglia-thalamocortical network contains 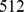 states, numbered from 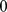 to 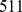. The numbering of the states is chosen such that state 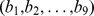 is numbered by 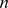, where 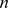 is the decimal representation of the 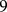-digit binary number 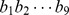. For instance, state 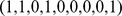 is referred to as number 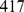, since 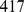 is the decimal representation of the binary number 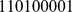. Secondly, also as mentioned in the proof of Proposition 4, the transitions of the Muller automaton 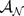 are constructed as follows: there is a transition labelled by 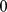 (resp. by 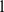) from state 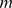 to state 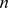 if and only if network 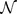 transits from state 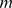 to state 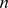 when it receives input 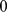 (resp. 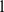). Hence, the deterministic Muller automaton 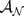 contains 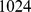 transitions (one 0-labelled and one 1-labelled outgoing transition from each of the 512 state), among which 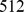 are labelled by 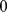 and 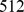 are labelled by 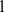. For instance, in the Muller automaton 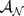 there is a transition labelled by 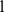 (drawn in red in Figure 8) from state 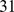 to state 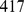 because network 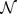 transits from state 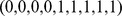 to state 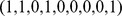 when it receives input 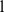. Figure 8a illustrates the graph of the deterministic Muller automaton associated to the basal ganglia-thalamocortical network.

An analysis of the graph of the automaton 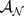 reveals that it contains only one strongly connected component 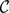 given by the states 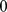, 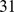, 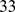, 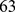, 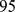, 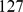, 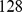, 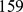, 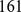, 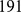, 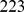, 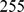, 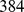, 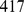, 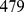, 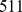 and the transitions between those states, as illustrated in Figure 8b (we recall that a directed graph is called strongly connected if there is a path from every vertex of the graph to every other vertex). This strongly connected component 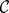 corresponds to the subgraph of 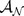 constituted by all states reachable from the initial state 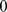. In other words, any state of 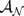 outside the strongly connected component 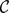 cannot be reached from the initial state 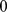, meaning that it can never occur in the dynamics of network 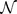 starting from initial state 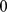, and hence plays no role in the attractor-based complexity of network 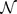. In fact, the attractor-based complexity of network 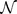 will be precisely determined by the cyclic structure of the strongly connected component 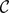 of automaton 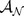.

In order to complete the description of the Muller automaton 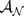, it is necessary to specify its table, or in other words, to determine among all possible cycles of 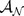 which ones are successful and which ones are non-successful. Since every cycle of 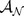 is by definition contained in a strongly connected component of 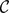 and since 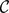 is the only strongly connected component of 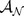, it follows that all cycles of 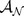 are necessarily contained in 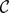. Therefore, the specification of the table of 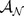 amounts to the assignment of a type specification to every cycle of the strongly connected component 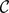. According to the biunivocal correspondence between cycles of 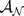 and attractors of 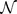, this assignment procedure consists in determining the type specification (meaningful or spurious) of all possible attractors of the network 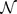.

In order to assign a type specification to every cycle of the strongly connected component 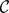, we have computed the list of all cycles starting from every state of 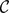, and for each cycle, we have further computed its decomposition into constitutive cycles (cycles which do not visit the same vertex two times). The results are summarised in Table 2.

**Table 2:** Number of cycles and constitutive cycles found for each starting state of the strongly connected component 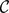.

State	# cycles	# constitutive cycles
0	68	24
31	47	20
33	87	24
63	93	21
95	39	21
127	21	17
128	63	24
159	77	22
161	72	20
191	52	19
223	43	21
255	53	17
384	67	24
417	35	20
479	48	16
511	84	21

**Figure 9:**
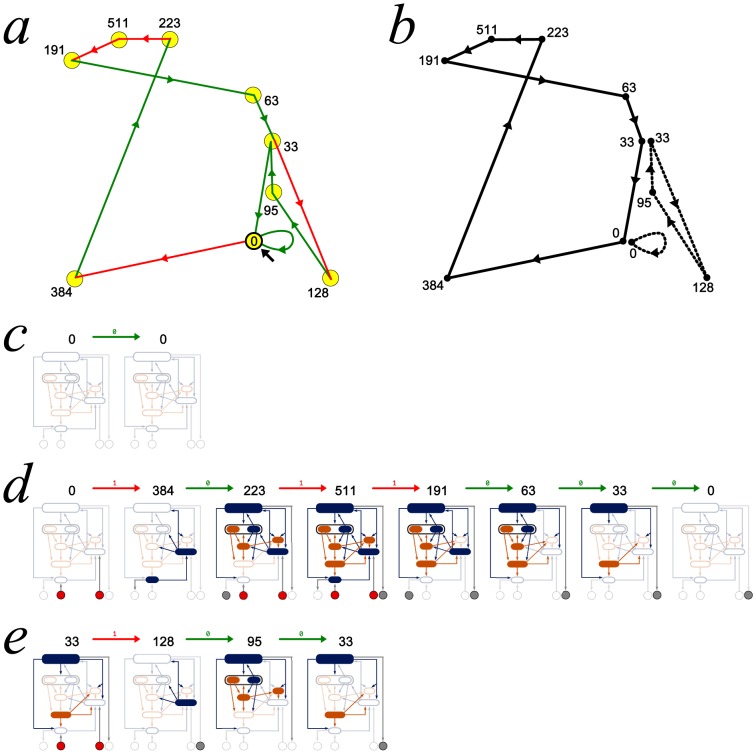
A cycle and its constitutive cycles. **a.** Among all cycles that can be observed starting from state 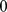 (indicated by the short arrow showing the entry point), we consider here an example, i.e. the cycle (0, 0, 384, 223, 511, 191, 63, 33, 128, 95, 33, 0). **b.** This cycle contains three constitutive cycles 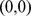, (0, 384, 223, 511, 191, 63, 33, 0) and (33, 128, 95, 33) that were assigned with type specification spurious (dotted line), meaningful (solid line), and spurious (dotted line), respectively. **c.** Sequence of states with graphical representation of the corresponding activated nodes of the basal ganglia-thalamocortical network for the spurious constitutive cycle 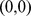. **d.** Sequence of states and activated network areas for the meaningful constitutive cycle (0, 384, 223, 511, 191, 63, 33, 0). **e.** Sequence of states and activated network areas for the spurious constitutive cycle (33, 128, 95, 33).

Then, we have assigned a type specification to each cycle of 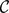 according to the following neurobiological criteria. First, a constitutive cycle is considered to be spurious if it is characterised either by active SC and quiet Thalamus at the same time step or by a quiet GPi/SNR during the majority of the duration of the constitutive cycle. A constitutive cycle is meaningful otherwise. Second, a non-constitutive cycle is considered to be meaningful if it contains a majority of meaningful constitutive cycles, and spurious if it contains a majority of spurious constitutive cycles – and in case of it containing as much meaningful as spurious constitutive cycles, its type specification was chosen to be meaningful. In order to illustrate this procedure, let us consider for example the cycles starting from state 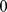. Table 2 shows that there are overall 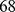 cycles and 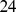 constitutive cycles starting from state 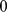. We consider here the example of one out of the 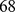 cycles, e.g. cycle 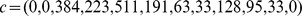 (Figure 9a). This cycle can be decomposed into three constitutive cycles (Figure 9b), namely 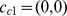, 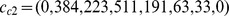, and 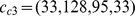. When state 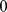 receives input 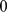 the network dynamics evolves into the constitutive cycle 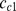 (Figure 9c), whereas if state 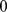 receives input 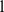 the dynamics evolves into the constitutive cycle 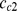 (Figure 9d). According to the aforementioned neurobiological criteria, the constitutive cycles 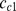 and 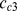 are spurious, whereas 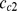 is meaningful, and therefore cycle 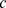 is spurious.

After the assignation of the type specification to every cycle, the attractor-based complexity of the network 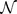 can be explicitly computed. More precisely, according to Theorem 6, the attractor-based degree of 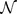 is given by the length of a maximal (co-)alternating tree contained in 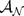. Since 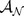 contains only one strongly connected component 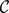, the maximal (co-)alternating tree of 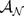 is necessarily contained in 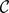. Indeed, every cycle of 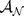 is, being a cycle, necessarily contained in a strongly connected component of 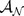; hence in particular, every cycle of the maximal (co-)alternating tree is also contained in a strongly connected component of 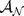; yet since 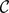 is the only strongly connected component of 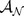, every cycle of the maximal (co-)alternating tree is contained in 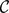, meaning that the maximal (co-)alternating tree itself is contained in 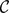.

**Figure 10:**
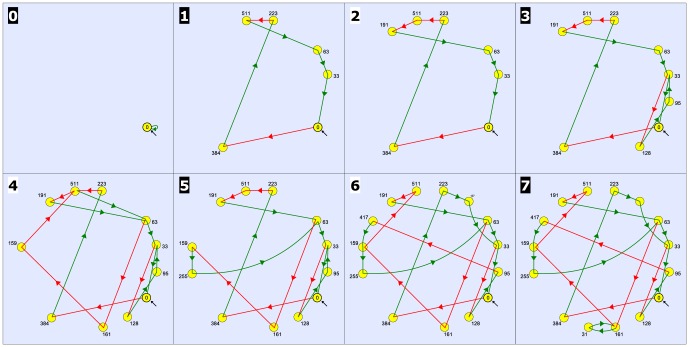
A maximal co-alternating tree of the deterministic Muller automaton 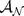. Panels 0 to 7 illustrate the sequence of eight cycles 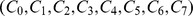 one included into the next. Cycles 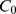, 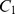, 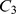, 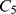, and 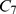 are spurious whereas cycles 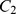, 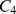, and 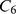 are meaningful. The sequence of cycles 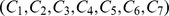 compose a maximal co-alternating tree of 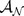. This maximal co-alternating tree contains 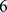 alternations between spurious and meaningful cycles, and thus has a length of 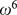. Therefore, the attractor-based degree of 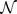 equals 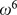.

After an exhaustive analysis of the strongly connected component 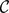 and of all its cycles (Table 2) we observed no maximal alternating trees with length above 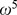. Conversely, we found 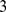 maximal co-alternating trees of 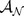 with length 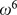. For sake of clarity, we describe one such maximal co-alternating tree: it consists of an alternating sequence of seven cycles included one into the other, summarised in Table 3 below and illustrated in Figure 10. Notice that there is no alternation between 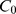 and 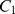 because both cycles *C*_0_ = (0, 0) and *C*_1_ = (0, 384, 223, 511, 63, 33, 0) are spurious. According to these results, it follows from Theorem 6 that the attractor-based complexity of network 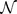 is 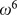 and that 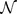 is non-self-dual.

**Table 3:** A maximal co-alternating tree of 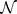 of length 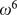 referred to Figure 10.

Name	State sequence	Specification type
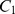	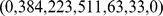	spurious
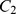	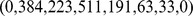	meaningful
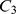	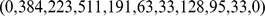	spurious
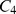	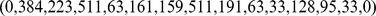	meaningful
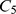	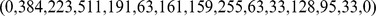	spurious
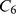	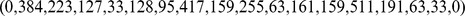	meaningful
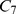	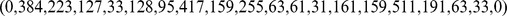	spurious

## Discussion

The present work revisits and extends in light of modern automata theory the seminal studies by McCulloch and Pitts, Minsky and Kleene concerning the computational power of recurrent neural networks [1]–[3]. We present two novel attractor-based complexity measures for Boolean neural networks, and finally illustrate the application of our results to a model of the basal ganglia-thalamocortical network.

More precisely, we prove two computational equivalence between Boolean neural networks and Büchi and Muller automata, and deduce from these results two hierarchical classifications of Boolean recurrent neural networks based on their attractive behaviours. The hierarchical classifications are obtained by translating the Wagner classification theory from the 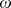-automaton to the neural network context. The first classification concerns the neural networks characterised by the specification of an output layer and the properties of the attractor dynamics associated with the activation of that output layer. In this case, the obtained hierarchical classification corresponds to a decidable pre-well ordering of width 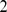 and height of 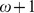. The second classification concerns the neural networks whose conditions on the type specifications of their attractors have been totally relaxed. In this case, the resulting hierarchy is significantly richer and consists of a decidable pre-well ordering of width 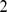 and height of 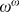. We prove that both hierarchical classifications are decidable and provide the decidability procedures aimed at computing the degrees of the networks in the respective hierarchies. We also show that the shorter hierarchy corresponds to an initial segment of the longer one in a precise sense. The notable result is the proof that the two hierarchical classifications are directly related to the attractive properties of the neural networks. More precisely, the degrees of the Boolean neural networks in the hierarchies correspond to the ability of the networks to maximally alternate between visits of meaningful and spurious attractors along their evolutions. The two hierarchies therefore provide two novel complexity measurments of Boolean recurrent neural networks according to their attractive potentialities. These complexity measurements represents an assessment of the computational power of Boolean neural networks in terms of the significance of their attractor dynamics.

### Attractor-Based Complexity Measurement

The degree of a neural network 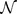 in the RNN hierarchy or in the complete RNN hierarchy corresponds precisely to the length of a maximal alternating chain or alternating tree contained in the graph of its corresponding automaton 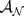, respectively. Since alternating chains and trees are described in terms of accessibility and inclusion relations between cycles of 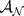, and according to the biunivocal correspondence between cycles of 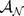 and attractors of 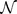, it follows that the degree of a neural network 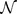 corresponds precisely to some intricacy relation – accessibility and inclusion – between the set of its meaningful and spurious attractors.

In order to better explain the attractor-based complexity measurement, suppose that some network 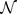 follows the periodic infinite evolution 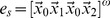, where 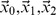 are states of 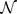. It follows that 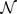 alternates infinitely often between the two cycles of states 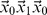 and 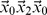, or equivalently, between the two attractors 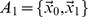 and 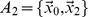. If we suppose that 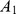 is meaningful and 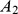 is spurious, then 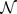 alternates infinitely often between a meaningful and a spurious attractor along the evolution 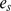. However, note that 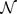 also visits infinitely often the composed attractor 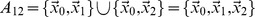. Hence, if 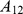 is meaningful (resp. spurious), then 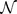 not only alternates infinitely often between a meaningful and a spurious attractor 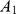 and 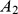 respectively, but also visits infinitely often the third composed meaningful (resp. spurious) attractor 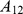.

In fact, for any infinite evolution 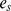, there always exists a unique such maximal attractor (maximal for the inclusion relation) that is visited infinitely often. Let us call this attractor the *global attractor* associated to 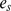. The attractor-based complexity measurement can now be understood as follows. A network 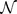 is more complex than a network 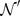 iff for any infinite evolution 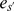 of 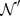, there exists a corresponding infinite evolution 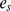 of 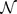 that can be build “simultaneously” to 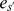 (in a precise sense described below) and such that, after infinitely many time steps, the types of global attractors visited by 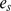 and 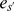 are the very same. In other words, a network 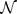 is more complex than a network 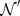 iff 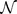 is able to mimic step by step every possible infinite evolution of 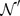 in order to finally obtain a global attractor of the same type.

This property can actually be precisely expressed in terms of game-theoretic considerations. Consider the game 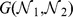 between networks 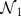 and 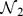 wholes rules are the following. Both networks begin in the rest state. Network 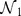 begins the game and 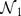 and 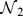 play in turn during infinitely many rounds. At every step, 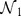 chooses a possible next state (accessible from its previous one), and 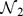 answers by either also choosing a possible next state (accessible from its previous one), or by skipping its turn. However, 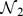 is obliged to chose infinitely many next states during the game. After infinitely many time steps, 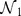 and 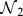 will have produced two infinite evolutions 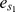 and 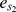, respectively. If the types of the global attractors of 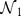 and 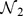 are the same, 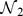 wins the game. Otherwise, 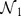 wins the game. One can prove that the attractor based complexity measures of 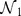 and 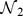 can then be expressed as follows: the degree of 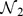 is higher than that of 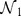 iff 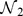 has a winning strategy in the game 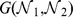.

In other words, a network 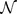 is more complex than 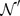 according to our attractor-based complexity iff 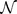 is capable of mimicking 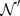 in every of its possible attractive behaviours. Two networks 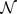 and 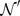 are equivalent if both are capable of mimicking each other in every one of its possible attractive behaviours. Assuming that the set of all possible attractive behaviours of a network is related to its computational power, our attractor-based complexity degree therefore represents a measurement of the computational power of Boolean neural networks in terms of the significance of their attractor dynamics.

Finally, note that the degree of a neural network in the RNN hierarchy or in the complete RNN hierarchy is intimately related to the structure of this network, more precisely to its connectivity. Indeed, for any neural network 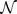 that would be given without any output layer or type specification of its attractors, it is possible to compute, by some graph analysis, the maximal alternating chains or alternating trees that could be contained in the graph of its corresponding automaton 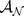, and therefore, by theorems 3 and 6, to know the maximal degree that this network could be able to achieve in the RNN or in the complete RNN hierarchy, if the type specification of its attractors were optimally distributed. In other words, any neural network, according to its connectivity structure, contains a potential maximal degree, which is achieved only if the set of its attractors are optimally discriminated into meaningful and spurious types. Hence, based on its connectivity, a certain network could be characterised by a high potential maximal degree, but in practice, due to a very limited discrimination – i.e. non-alternation – between its spurious and meaningful attractors, it will exhibit a low degree of network complexity.

### Significance of measuring network complexity

In an application of our network complexity measurement to a model of a real brain circuit, we demonstrated that, under specific assumptions of connectivity and dynamics, the basal ganglia-thalamocortical network can be modeled by a network of degree 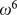. Why is it so interesting to know this degree? What kind of increased understanding of that network do we gain from that? The degree of network complexity for a given network is important to be determined if we want to assess the computational power that can be achieved by that network. In other words, the degree of network complexity is a functional characteristic of a given network.

For example, a model of the basal ganglia-thalamocortical network with a complexity of degree 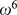 is able to perform all possible computations made by a model of the same network with a complexity of degree 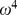 and many more computations in addition. If we were able to associate certain functional states of cognitive relevance (or certain pathological conditions of clinical relevance, respectively) to an increase (or to a decrease, respectively) in network complexity, we would certainly gain a better insight into the role and the factors that modulate the operations executed by certain brain circuits.

Then, how and why the network complexity of a model of the basal ganglia-thalamocortical network could vary? The degree of complexity of a network is upper bounded by its potential maximal degree. In the next section, we discuss how control parameters can affect network dynamics and eventually its complexity degree.

### Control parameters of network dynamics

The central hypothesis for brain attractors is that, once activated by appropriate activity, the network behaviour is maintained by continuous reentry of activity, thus generating a high incidence of repeating firing patterns associated with underlying attractors [37], [38]. The question whether the attractors revealed by certain patterns of activity are spurious or meaningful cannot be answered easily. Certain patterns may repeat above chance and occur transiently during the evolution of a network [23], [55] and during the transient inactivation of part of the newtwork, as shown experimentally with thalamic firing patterns during reversible inactivation of the cerebral cortex [60], [107]. On the other hand, patterns and attractors *per se* may reveal an epiphenomenon or a byproduct of the network dynamics, thus being classified as spurious. However, changing conditions and association of attractors into higher-order attractors may turn a spurious into a meaningful type, and vice versa. For this reason, in the present paper, we have emphasised the importance of the specification types of the constitutive cycles and how these affect the specification type of a cycle.

The measurements of networks complexities refer to the possibility of networks' dynamics to maximally alternate between attractors of different types along their evolutions. This is interesting for an overall assessment of the properties of a network because it associates the computational power of that network with the significance of their attractor dynamics.

The excitatory/inhibitory balance in a neural network is the major factor affecting the dynamics of its activity [38], [109]–[111]. The activity of the basal ganglia-thalamocortical network is modulated by a complex set of brain structures, including the dopaminergic (including those from the substantia nigra pars compacta like the nigrostriatal and those from the ventromedial tegmental area), cholinergic (including those from the basal forebrain), the noradrenergic (including those from locus coeruleus), serotoninergic (including those from the dorsal raphe), histaminergic (from the tuberomamillary nucleus) and orexinergic nuclei (from the lateral and posterior hypothalamus) [103], [112]–[115]. These neuromodulators affect, among other parameters, the synaptic kinetics (i.e., the decay time of the synaptic interaction) and the cellular excitability, thus producing stable or unstable spatiotemporally organised modes of activity and rapid state switches [69], [111], [116]–[119]. The effect of cholinergic modulation exerted by the basal forebrain is particularly noticeable to this aspect [120]–[122].

**Figure 11:**
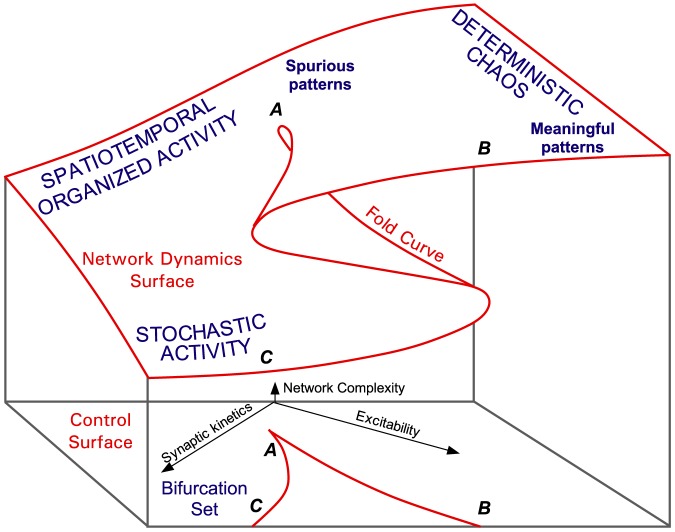
Cusp catastrophe model. We consider an example of network dynamics controlled by two independent parameters, the synaptic kinetics and the cell excitability. Divergent behaviour is accounted for since as the dynamics moves out from the edge (point A) toward the fold, which is the starting point of separation between an upper and lower limbs, the network dynamics is forced to move towards one of the two opposing behaviours: either point B for network dynamics dominated by deterministic chaos and chaotic itinerancy, or point C for network dynamics dominated by stochastic activity.

The possible different dynamics of a given network can be represented by an equilibrium surface where each point is determined by a network complexity associated with two (in the simplest abstraction) independent variables. Such a situation is illustrated in Figure 11 by the cusp catastrophe of the Catastrophe theory [108]. In our example, the two control parameters are the excitability and the synaptic kinetics. Depending on the ranges of the parameters that control the network dynamics, the network complexity may remain identical or only slightly modified, in which case we refer to a “smooth” path on the network dynamics surface. In other cases, small changes in the parameter values may provoke rapid state switches corresponding to “sudden” changes in network complexity (e.g., see [111]).

The network dynamics surface has a singularity represented by a fold (or Riemann-Hugoniot cusp) in it. A bifurcation set is defined by the thresholds where sudden changes can occur, depending on the initial conditions, by projecting the cusp onto the control surface. The network complexity, as defined in this study, depends on the maximal (co-)alternation between spurious and meaningful attractors. In the network dynamics surface, the edge toward the fold (point A, in Figure 11) is the starting point of separation between two surfaces. One surface is the top sheet representing network dynamics with a high degree of complexity because of the presence of deterministic chaos enabling the possibility to increase the (co-)alternation by mean of chaotic itinerancy (point B, in Figure 11) [66], [67], [69]. The other surface is the bottom sheet reflecting the dominance of stochastic dynamics, hence absence of alternation (point C, in Figure 11). Hence, as the network dynamics moves out from the edge near the fold the dynamics is diverging and forced to move toward one of the two opposing behaviours. The path that will be followed by the dynamics depends on the values of the control parameters defining the state of the neural network just prior to reaching the fold. Sudden transitions are accounted for at the edges of the fold, for example as the stochastic dynamics moves along the surface toward the pleat, at some point a small change in control parameters may cause a sudden shift such that, after a long interval without cyclic activity, quasi-random activity develops into quasi-attractors and long cycles may suddenly appear containing many constitutive cycles and many alternations between spurious and meaningful attractors, e.g. tuning thalamic activity by corticofugal activity [123], [124].

### Conclusion

The present work can be extended in at least three directions. First, it is expected to study the computational and dynamical complexity of neural networks induced by other mathematical bio-inspired criteria. Indeed, the approach followed in this paper provides a hierarchical classification of neural networks according to the topological complexity of their underlying neural languages, and subsequently, according to the complexity of their attractive behaviours. However, it remains to be clarified how this natural mathematical criterion could be translated into the real biological complexity of the networks. Other complexity measures might bring further insights to the global understanding of brain information processing.

Secondly, it is envisioned to describe the computational power of more biologically oriented neuronal models. For instance, first-order recurrent neural networks provided with some simple spike-timing dependent plasticity (STDP) rule could be of interest [48], [125]–[128]. Also, neural networks equipped with more complex activation function or dynamical equations governing the membrane dynamics could be relevant [129]. Important preliminary steps in this direction were made by providing a description of the computational capabilities of static/evolving rational-weighted/analog recurrent neural networks involved in a classical as well as in a memory active and interactive paradigm of computation [6], [11],[27],[31]–[33].

The third and maybe most important extension of our study is oriented towards the application of our new attractor-based complexity measurement to other examples of real neural networks, and to studying the effect of modulatory projections in controlling the network complexity. Indeed, the parameters that control neural dynamics (e.g., excitability and synaptic kinetics) are driven by so-called modulatory projections, such as the cholinergic and serotoninergic projections.

Finally, we believe that the theoretical approach to the computational power of neural models might ultimately bring further insight to the understanding of the intrinsic natures of both biological as well as artificial intelligences. On the one hand, the study of the computational and dynamical capabilities of brain-like models might improve the understanding of the biological features that are most relevant to brain information processing. On the other hand, foundational approaches to alternative models of computation might lead in the long term not only to relevant theoretical considerations [130], [131], but also to practical applications.

## Supporting Information

File S1
**Example S1,** Description of a deterministic Büchi automaton, and illustration of the concept of an alternating chain. **Figure S1, A deterministic Büchi automaton** 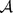 **.** The nodes and edges correspond to the states and transitions of 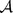, respectively. The node 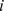 corresponds to the initial state, as indicated by the short input arrow. The double-circled red nodes correspond to the final states of 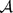. The Büchi automaton 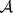 contains a maximal alternating chain of length 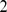, and a maximal co-alternating chain of length 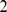 also.(ZIP)

File S2
**Example S2,** Description of a deterministic Muller automaton, and illustration of the concept of an alternating tree. **Figure S2, A Muller automaton** 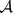 **.** The underlying alphabet of 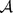 is 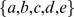. The table 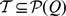 represents the set of cycles of 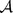 that are successful. All other cycles of 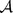 are by definition non-successful. The successful and non-successful cycles are denoted in green and red, respectively. This Muller automaton 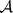 contains a maximal alternating tree of length 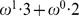.(ZIP)

File S3
**Example S3,** Illustration of the translation procedures described in Propositions 1 and 2. **Figure S3, Panels a, b.** Translation from a neural network to its corresponding deterministic Büchi automaton. **a.** The neural network 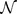 of Figure 1 provided with an additional specification of an output layer 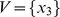 denoted in red and double-circled. **b.** The deterministic Büchi automaton 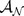 corresponding to the neural network 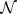 of panel *a*. The final states are denoted in red and double-circled, and the active status of the output layer, namely cell 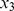, is emphasised by a bold red 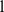. **Panels c, d.** Translation from a deterministic Büchi automaton to its corresponding neural network. **c.** A deterministic Büchi automaton 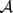 with three states. The initial state 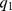 is denoted with an incoming edge. The final state 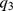 is emphasised in red and double-circled. **d.** The network 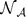 corresponding to the Büchi automaton 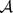. The output layer is represented by the cell 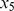, denoted in red and double-circled. The background activities are labeled in blue.(ZIP)

File S4 **Example S4,** Illustration of the decidability procedure of the RNN hierarchy.
(ZIP)

File S5
**Example S5,** Illustration of the translation procedures described in Propositions 4 and 5. **Figure S4, Panels a, b.** Translation from a Boolean neural network provided with a type specification of its attractors to its corresponding deterministic Muller automaton. **a.** A neural network 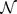 provided with an additional type specification of each of its attractors. In this case, 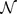 contains only one meaningful attractor determined by the following set of states 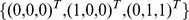; all other ones are considered as spurious. **b.** The deterministic Muller automaton 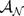 corresponding to the neural network 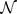 of panel *a*. Automaton 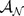 works over alphabet 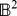, contains six states, and possesses in its table 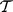 the sole cycle 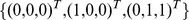, which corresponds to the sole meaningful attractor of 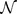. **Panels c, d.** Translation from a deterministic Muller automaton to its corresponding Boolean neural network provided with a type specification of its attractors. **c.** A deterministic Muller automaton 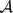. The automaton works over alphabet 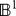, has three states, and possesses the two successful cycles 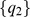 and 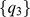, as mentioned by its table 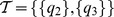. **d.** The neural network 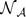 corresponding to the Muller automaton 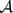 of panel *c*. The network 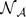 contains two letter cells, one delay cell, and three state cells to simulate the two possible inputs and three states of automaton 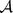. It has only two meaningful attractors corresponding to the two successful cycles of automaton 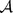.(ZIP)

File S6 **Example S6,** Illustration of the decidability procedure of the complete RNN hierarchy.
(ZIP)
