# Supplementary figures and images for: An Attractor-Based Complexity Measurement for Boolean Recurrent Neural Networks
Source: PLoS One. 2014 Apr 11;9(4):e94204. doi: 10.1371/journal.pone.0094204 (PMC3984152; doi:10.1371/journal.pone.0094204)

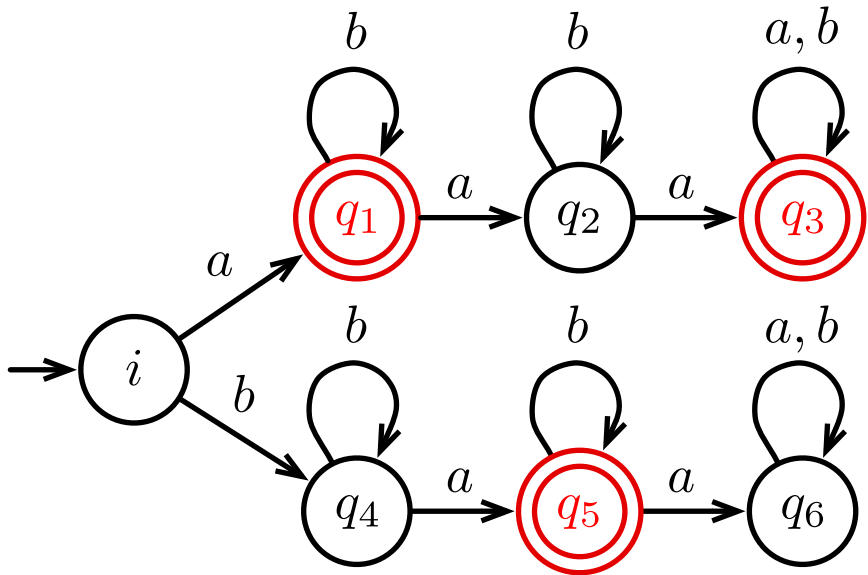

Supplement: File S1 — Example S1, Description of a deterministic Büchi automaton, and illustration of the concept of an alternating chain. Figure S1, A deterministic Büchi automaton . The nodes and edges correspond to the states and transitions of , respectively. The node corresponds to the initial state, as indicated by the short input arrow. The double-circled red nodes correspond to the final states of . The Büchi automaton contains a maximal alternating chain of length , and a maximal co-alternating chain of length also. (ZIP) [file pone.0094204.s001.zip › figS1n-Buchi.pdf]

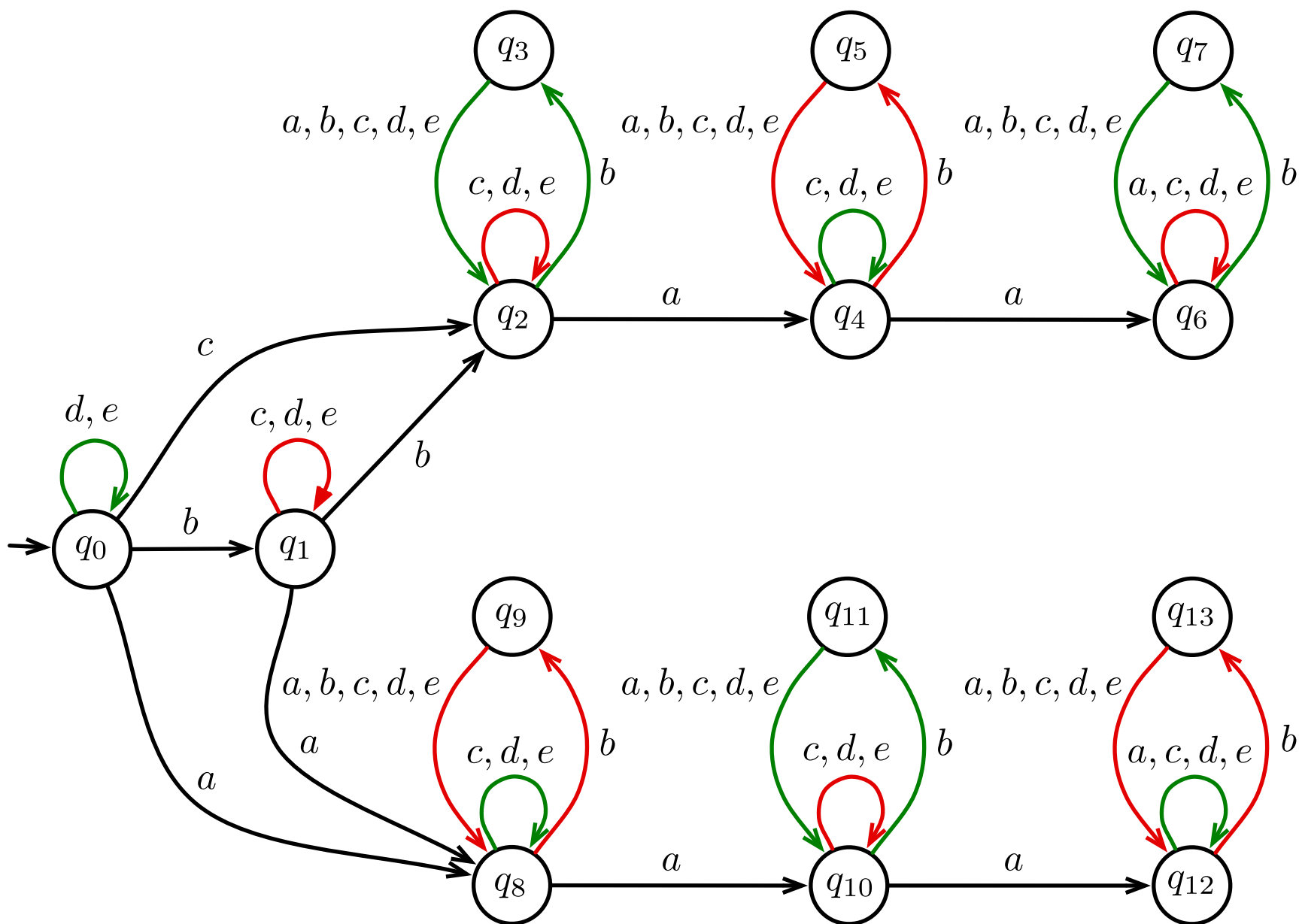

$$\mathcal{T} = \{\{q_0\}, \{q_2, q_3\}, \{q_4\}, \{q_6, q_7\}, \{q_8\}, \{q_{10}, q_{11}\}, \{q_{12}\}\}$$

Supplement: File S2 — Example S2, Description of a deterministic Muller automaton, and illustration of the concept of an alternating tree. Figure S2, A Muller automaton . The underlying alphabet of is . The table represents the set of cycles of that are successful. All other cycles of are by definition non-successful. The successful and non-successful cycles are denoted in green and red, respectively. This Muller automaton contains a maximal alternating tree of length . (ZIP) [file pone.0094204.s002.zip › figS2n-Muller.pdf]

*a*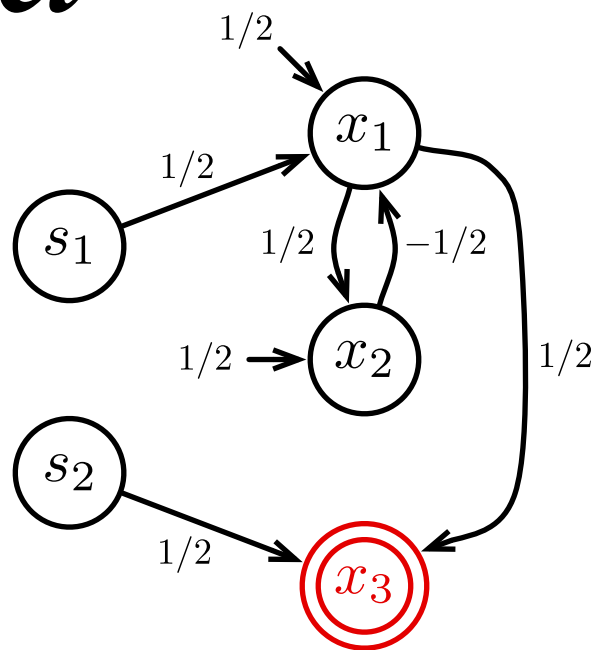*b*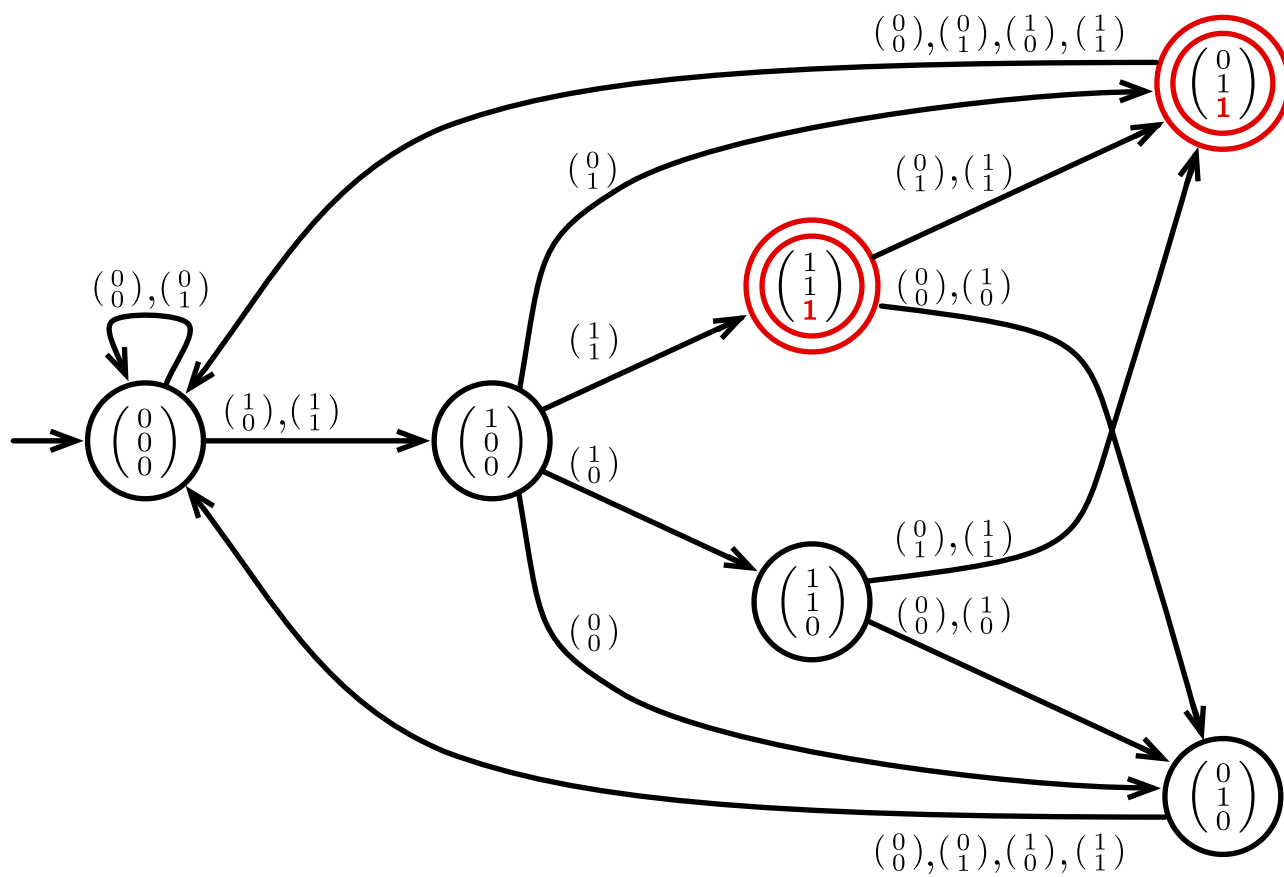*c*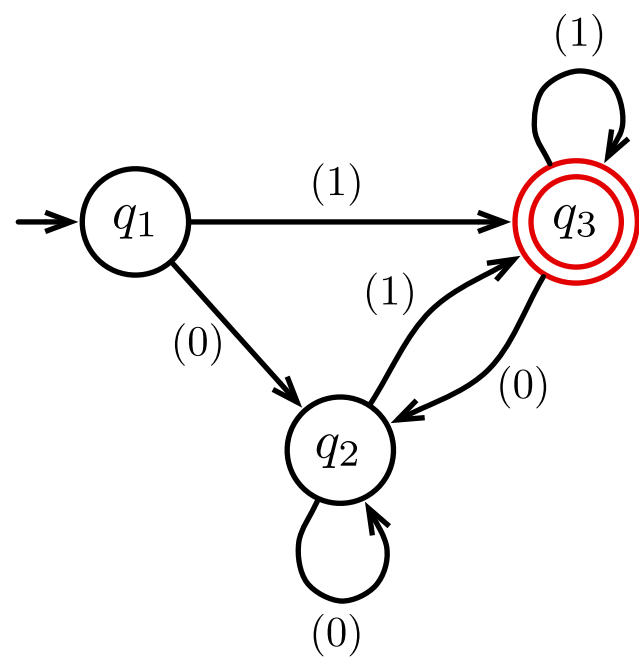*d*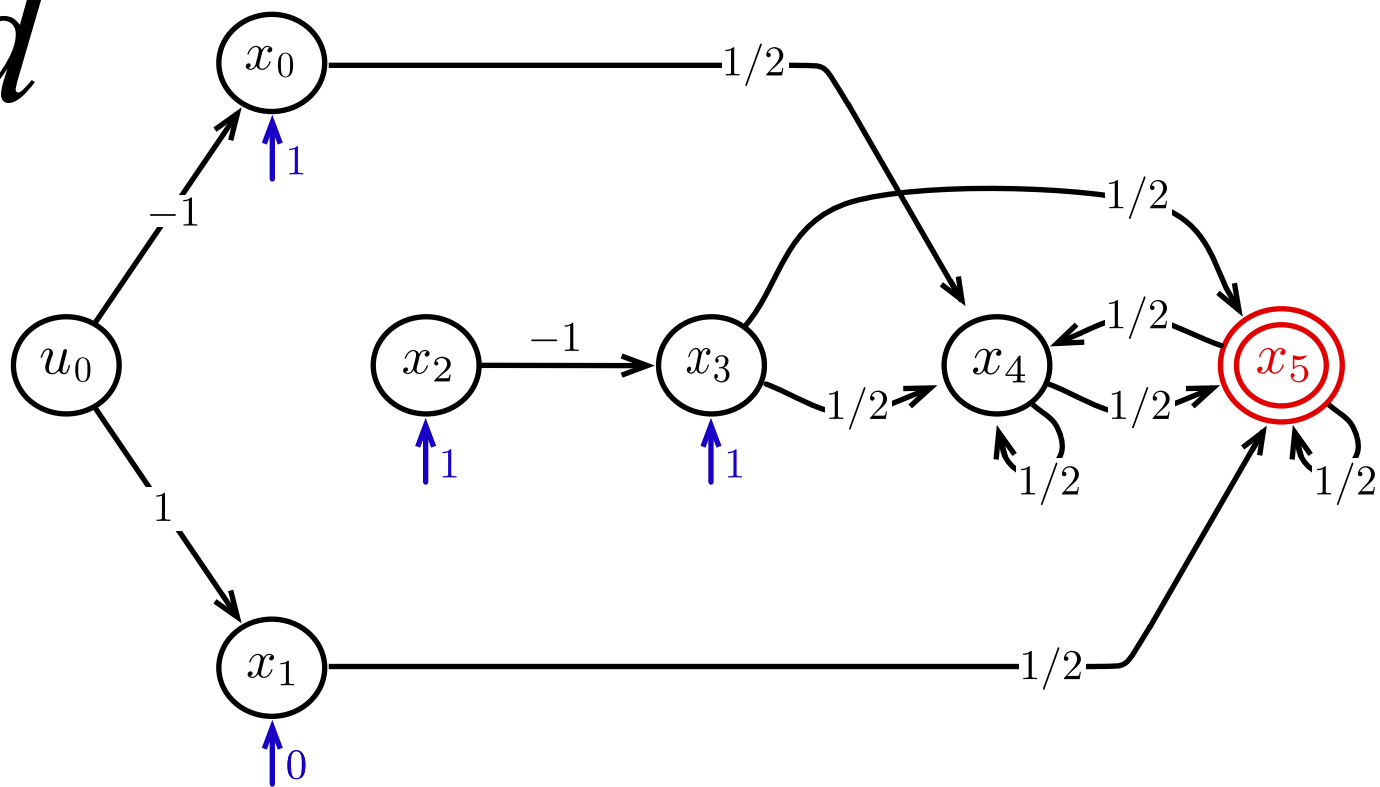

Supplement: File S3 — Example S3, Illustration of the translation procedures described in Propositions 1 and 2. Figure S3, Panels a, b. Translation from a neural network to its corresponding deterministic Büchi automaton. a. The neural network of Figure 1 provided with an additional specification of an output layer denoted in red and double-circled. b. The deterministic Büchi automaton corresponding to the neural network of panel a. The final states are denoted in red and double-circled, and the active status of the output layer, namely cell , is emphasised by a bold red . Panels c, d. Translation from a deterministic Büchi automaton to its corresponding neural network. c. A deterministic Büchi automaton with three states. The initial state is denoted with an incoming edge. The final state is emphasised in red and double-circled. d. The network corresponding to the Büchi automaton . The output layer is represented by the cell , denoted in red and double-circled. The background activities are labeled in blue. (ZIP) [file pone.0094204.s003.zip › figS3n-directionB.pdf]
