# Supplementary material for: An Attractor-Based Complexity Measurement for Boolean Recurrent Neural Networks
Source: PLoS One. 2014 Apr 11;9(4):e94204. doi: 10.1371/journal.pone.0094204 (PMC3984152; doi:10.1371/journal.pone.0094204)
Supplement: File S5 — Example S5, Illustration of the translation procedures described in Propositions 4 and 5. Figure S4, Panels a, b. Translation from a Boolean neural network provided with a type specification of its attractors to its corresponding deterministic Muller automaton. a. A neural network provided with an additional type specification of each of its attractors. In this case, contains only one meaningful attractor determined by the following set of states ; all other ones are considered as spurious. b. The deterministic Muller automaton corresponding to the neural network of panel a. Automaton works over alphabet , contains six states, and possesses in its table the sole cycle , which corresponds to the sole meaningful attractor of . Panels c, d. Translation from a deterministic Muller automaton to its corresponding Boolean neural network provided with a type specification of its attractors. c. A deterministic Muller automaton . The automaton works over alphabet , has three states, and possesses the two successful cycles and , as mentioned by its table . d. The neural network corresponding to the Muller automaton of panel c. The network contains two letter cells, one delay cell, and three state cells to simulate the two possible inputs and three states of automaton . It has only two meaningful attractors corresponding to the two successful cycles of automaton . (ZIP) [file pone.0094204.s005.zip › figS4n-directionM.pdf]

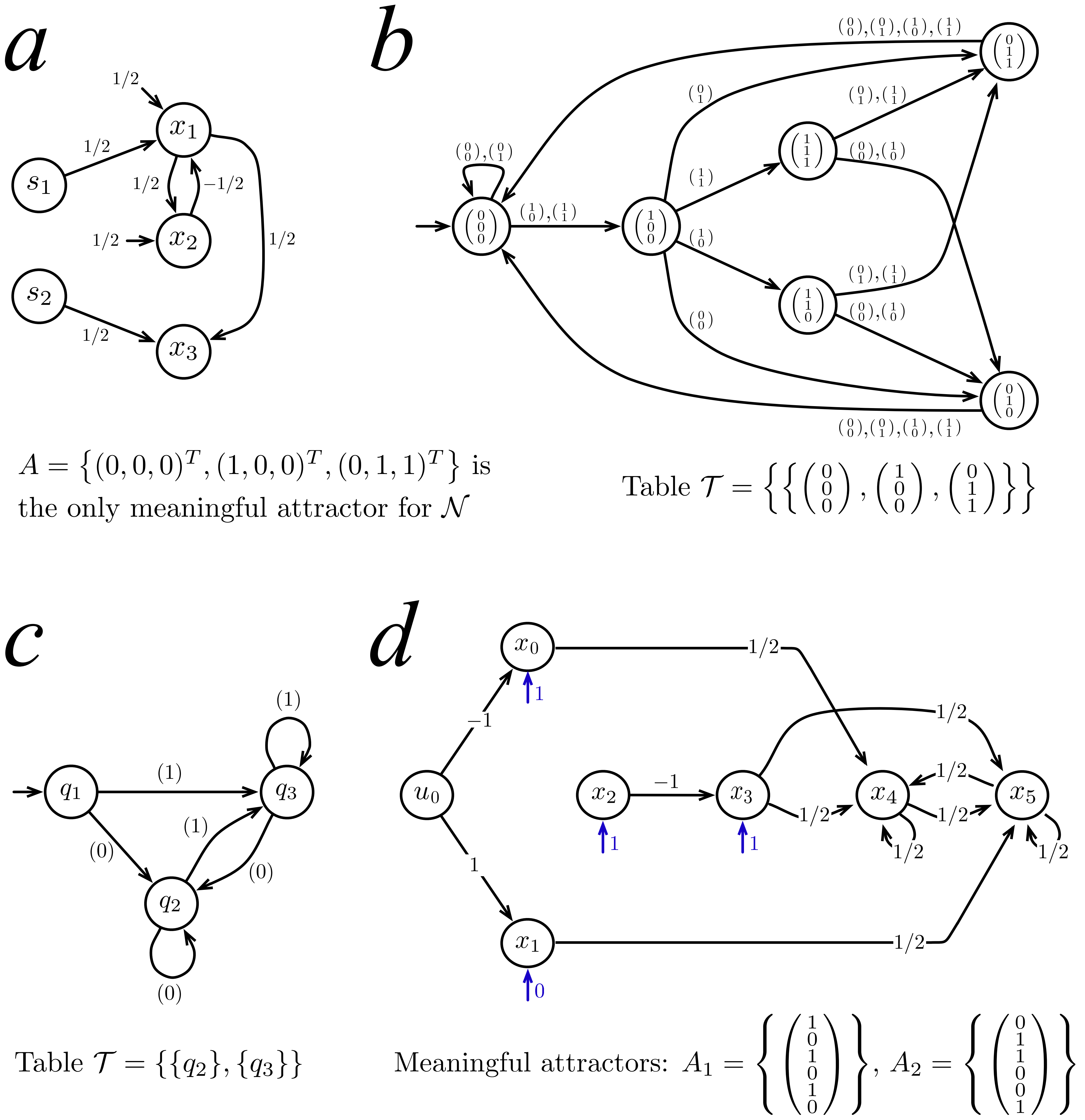

a
1/2
x_1
1/2
s_1
1/2
−1/2
x_2
1/2
1/2
s_2
x_3
1/2
$A = \{(0,0,0)^T, (1,0,0)^T, (0,1,1)^T\}$ is the only meaningful attractor for $\mathcal{N}$
b
Table $\mathcal{T} = \left\{\left\{\begin{pmatrix}0\\0\\0\end{pmatrix}, \begin{pmatrix}1\\0\\0\end{pmatrix}, \begin{pmatrix}0\\1\\1\end{pmatrix}\right\}\right\}$
c
(1)
(1)
q_1
q_3
(0)
(1)
(0)
q_2
(0)
Table $\mathcal{T} = \{\{q_2\}, \{q_3\}\}$
d
x_0
1/2
1
−1
1/2
u_0
x_2
−1
x_3
x_4
1/2
x_5
1/2
1/2
1
1
1/2
1/2
1
x_1
1/2
0
Meaningful attractors: $A_1 = \left\{\begin{pmatrix}1\\0\\1\\0\\1\\0\end{pmatrix}\right\}$, $A_2 = \left\{\begin{pmatrix}0\\1\\1\\0\\0\\1\end{pmatrix}\right\}$
